# Machine Learning-Based Hazard-Driven Prioritization of Features in Nontarget Screening of Environmental High-Resolution Mass Spectrometry Data

**DOI:** 10.1021/acs.est.3c00304

**Published:** 2023-06-06

**Authors:** Katarzyna Arturi, Juliane Hollender

**Affiliations:** † Department of Environmental Chemistry, Swiss Federal Institute of Aquatic Science and Technology (Eawag), Ueberlandstrasse 133, 8600 Dübendorf, Switzerland; ‡ Institute of Biogeochemistry and Pollution Dynamics, Eidgenössische Technische Hochschule Zürich (ETH Zurich), Rämistrasse 101, 8092 Zürich, Switzerland

**Keywords:** ToxCast, Tox21, toxicity prediction, HRMS/MS, supervised classification, extreme gradient boosting, SIRIUS

## Abstract

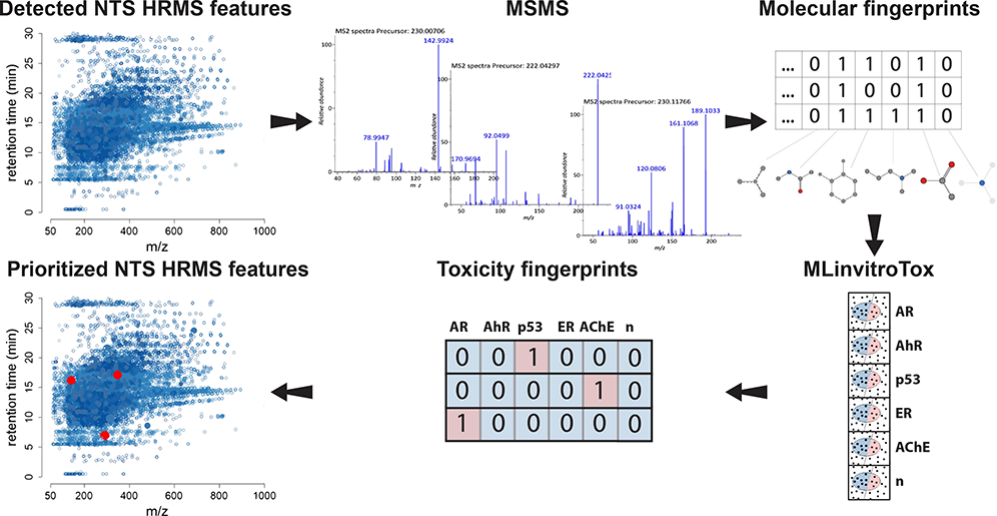

Nontarget high-resolution mass spectrometry screening (NTS HRMS/MS) can detect thousands of organic substances in environmental samples. However, new strategies are needed to focus time-intensive identification efforts on features with the highest potential to cause adverse effects instead of the most abundant ones. To address this challenge, we developed MLinvitroTox, a machine learning framework that uses molecular fingerprints derived from fragmentation spectra (MS2) for a rapid classification of thousands of unidentified HRMS/MS features as toxic/nontoxic based on nearly 400 target-specific and over 100 cytotoxic endpoints from ToxCast/Tox21. Model development results demonstrated that using customized molecular fingerprints and models, over a quarter of toxic endpoints and the majority of the associated mechanistic targets could be accurately predicted with sensitivities exceeding 0.95. Notably, SIRIUS molecular fingerprints and xboost (Extreme Gradient Boosting) models with SMOTE (Synthetic Minority Oversampling Technique) for handling data imbalance were a universally successful and robust modeling configuration. Validation of MLinvitroTox on MassBank spectra showed that toxicity could be predicted from molecular fingerprints derived from MS2 with an average balanced accuracy of 0.75. By applying MLinvitroTox to environmental HRMS/MS data, we confirmed the experimental results obtained with target analysis and narrowed the analytical focus from tens of thousands of detected signals to 783 features linked to potential toxicity, including 109 spectral matches and 30 compounds with confirmed toxic activity.

## Abstract 2

MLinvitroTox maps toxicologically relevant pollution in aquatic environments by predicting the toxicity of unidentified NTS HRMS/MS features from fragmentation spectra via machine learning.

## Introduction

Environmental pollution, fueled by growing chemical production and discharge of domestic, agricultural, and industrial wastes, significantly increased in the 20th century affecting biodiversity and causing contamination of the food chains and lack of potable water. While more than 200 million compounds have been registered by Chemical Abstracts Service (CAS) to date and an estimated 30,000–70,000 chemical species are used in households alone, only a few hundred are monitored worldwide via target analytical approaches.^1^ Advances in modern analytical methods such as high-resolution mass spectrometry (HRMS/MS) reveal that thousands of anthropogenic pollutants with poorly understood toxicological properties are released to the aquatic environments daily.^2^ To map the complexity of global pollution, sophisticated nontarget screening (NTS) data processing workflows^3−7^ have been developed for HRMS/MS. These approaches employ numerous computational and machine learning (ML) tools^8−18^ to identify and quantify novel organic pollutants in the environment based on their MS1 (abundance of parent ions) and MS2 (fragmentation spectra). Although thousands of molecular HRMS/MS features in complex aquatic mixtures can be routinely discerned and processed via NTS, a complete elucidation (unequivocal identification and quantification) of a large number of signals is not yet feasible due to the necessity of manual validation and confirmation with reference standards.^19^ Most commonly, the unidentified features are prioritized in NTS based on the intensity of the measured signal as a proxy of abundance. Given that intensity does not necessarily reflect the concentration,^17^ the conventional prioritization strategy fails to capture the environmental exposures of unknown compounds. Moreover, it also lacks a toxicological element, thus entirely disregarding environmental risks (= exposure × hazard) of emerging pollutants. For example, endocrine-disrupting compounds^20^ and pyrethroids,^21^ which are extremely harmful to aquatic organisms at very low (10^–12^ g/L) levels, would not be revealed with the abundance-based prioritization.

Establishing toxicological relevance in HRMS/MS analysis is a complex task. So far, only a few hundred chemicals have been comprehensively studied due to the time-consuming, expensive, and ethically questionable nature of the traditional *in vivo* toxicity testing on animals.^22^ So even if an emerging pollutant can be identified, its toxicity data is most likely unavailable. Even fewer resources exist to assess the toxicity of complex environmental samples revealed by NTS HRMS/MS. Efforts have been extended to link composition (from HRMS/MS) to the toxic effect of complex mixtures via effect-directed analysis (EDA),^23,24^ which aims at a deductive identification of the compounds in sample fractions with a particular toxic outcome. Toxicity in EDA is most often assessed via *in vitro* bioassays on, e.g., cell cultures for indirectly evaluating hazard potentials by focusing on single cellular mechanisms instead of overall toxic outcomes.^22^ To decrease the manual workload during EDA, high throughput (HT-EDA) was developed in recent years.^25^ However, not all assays can be used in the HT-EDA mode. Furthermore, due to the low potency and abundance of the majority of unidentified HRMS/MS features, HT-EDA is unsuitable for processing thousands of NTS signals typically detected in complex matrices.

In high throughput screening (HTS), thousands of *in vitro* bioassays can be conducted with a combination of robotics, automated analysis, and data processing, giving rise to high-volume toxicity data. Since the publication of the landmark report *Toxicity Testing in the 21st Century: Vision and Strategy* in 2007 by the U.S. National Academy of Sciences,^22^ a major paradigm shift in toxicity testing resulted in the development of HTS toxicity databases such as ToxCast and Tox21 (invitroDB^26,27^). While the primary aim of HTS was to replace *in vivo* studies on animals with *in silico* and mechanistic studies, it also opened new exciting opportunities for ML-based predictive computational toxicology, particularly for predicting toxicity from structure via molecular fingerprints with QSARS (Quantitative Structure–Activity Relationships). Molecular fingerprints are mathematical representations of molecules encoding the structures as binary vectors of fixed length where each bit describes the presence (1) or absence (0) of a particular substructure. Using *in vitro* data for predicting toxicity is based on the assumption that molecular toxic effects are activated by relatively simple interactions between specific chemical moieties and, e.g., a receptor during a molecular initiating event (MEI) starting a series of key events (KE) in cells that may lead to an adverse outcome pathway (AOP) on the organ or organism level. According to a recent review,^28^ 542 papers utilizing invitroDB have been published since 2006, covering topics such as toxic potential of chemicals, identification of contaminants for environmental monitoring, and computational toxicity prediction. The majority of invitroDB-based ML applications developed to date focused on relatively few target-specific endpoints and cytotoxicity.^29−45^ Endocrine receptor systems, in particular, androgen and estrogen receptors, as well as carcinogenicity, hepatic steatosis, hepatotoxicity, immunotoxicity, developmental toxicity, neurotoxicity, and cardiotoxicity were the most widely studied adverse outcomes.^46^

**Figure 1:**
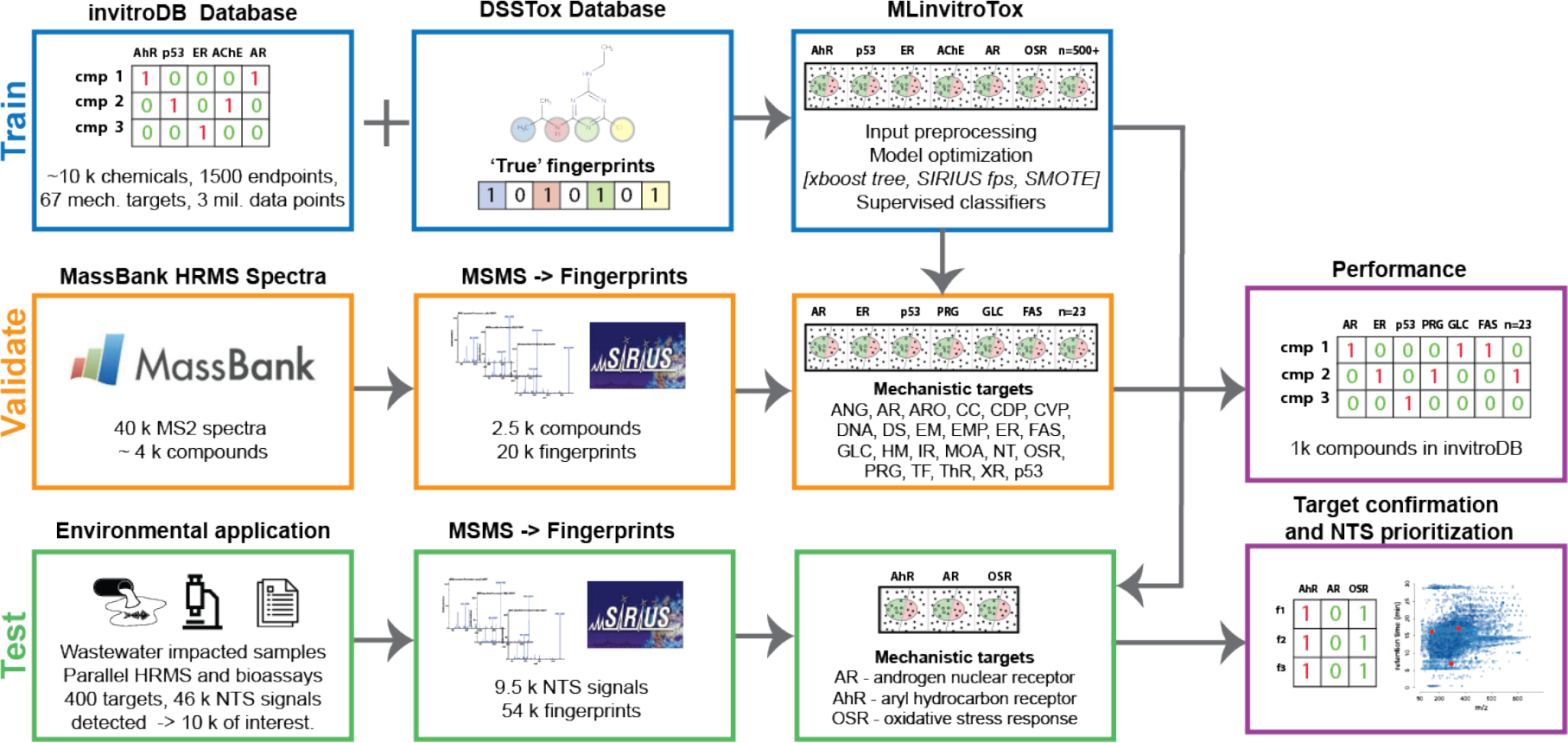
Workflow for developing and validating machine learning-based prediction of molecular toxicity for unidentified HRMS/MS features. MLinvitroTox models were trained on invitroDB, validated using the MassBank spectral library, and tested on environmental HRMS/MS measurements from Neale et al.^51^

ML-based toxicity prediction has shown potential, as demonstrated by the successes of the Tox21 Data Challenge 2014.^47^ However, low transferability and a lack of mechanistic model interpretation have constrained this approach’s widespread use in adjacent scientific fields. In this work, we developed a hazard-driven prioritization of unidentified NTS HRMS/MS signals based on ML-based toxicity prediction to improve the mapping of toxicologically relevant pollution in aquatic environments. Unlike traditional QSARs that rely on predicting activities based on molecular fingerprints derived from structures, MLinvitroTox was trained on structures but applied to molecular fingerprints predicted from the experimentally measured MS2 spectra via CSI:FingerID/SIRIUS.^15^ SIRIUS is a software package for annotating small molecules from nontarget HRMS/MS. CSI:FingerID is a machine-learning tool SIRIUS uses to predict molecular fingerprints from fragmentation spectra. Compared to similar efforts in the field where ecotoxicity was predicted from MS2 based on *in vivo* data,^48−50^ in the current work, the invitroDB toxicity database was used to train supervised classification models for hundreds of available toxicity endpoints ensuring broad toxicological coverage of the combined ML framework, termed **MLinvitroTox**. Modeling of previously unexplored invitroDB endpoints was in the current work enabled by developing a custom curation of structural and toxicological data designed to address challenges from modeling dirty, sparse, and imbalanced data sets. Extensive development of MLinvitroTox was followed by validation with MassBank spectral library and testing on environmental samples studied previously,^51^ including a mechanistic elucidation of the ML models in terms of structural moieties’ contribution to toxicity. Figures 1 and SF1 conceptualize MLinvitroTox.

## Material and Methods

### Toxicity Data

For toxicity modeling, the high-throughput screening (HTS) *in vitro* toxicity database (invitroDB v3.3, 10.23645/epacomptox.6062623.v6,^27^https://www.epa.gov/chemical-research/exploring-toxcast-data-downloadable-data) from the Environmental Protection Agency (U.S. EPA) was downloaded as MySQL and installed locally. The invitroDB data spanned nearly 800 HTS bioassays covering 1473 molecular toxicity endpoints tested selectively across more than 10k chemicals, resulting in more than 3.72 million toxicity data points. Each point represented a unique chemical/endpoint pair and was associated with a particular toxic outcome (expressed with specific points of departure [e.g., AC50 is the concentration of a chemical producing 50% of the maximal effect]) and a binary toxicity call [hitc = 1 for toxic and hitc = 0 for nontoxic]), as well as metadata such as fitted models, parameters, warning flags, fit categories, and uncertainty estimates. The toxicity data covered a range of high-level cell responses corresponding to nearly 70 mechanistic targets and more than 300 signaling pathways associated with nearly 400 AOPs linking the molecular activities to adverse effects on the organ or organism level. The data were processed in R (v4.0.3.) with the standardized data analysis pipeline tcpl R package.^52^ In short, multiple concentration toxicity data was used to build dose–response curves with baseline median absolute deviation (BMAD) as three times the median absolute deviation (3·MAD) and ACC (concentration at the user-defined cutoff value) computed at 6·BMAD. Three models were fit (constant, gain-loss, and hill), a winning model was chosen based on AIC (Akaike Information Criterion), and corresponding points of departure (e.g., AC50 and ACC) were calculated. A dose–response series was assigned an active (toxic) hit-call label (hitc = 1) when either the hill or gain-loss was the winning model and both the modeled curve fit top (modl_tp) as well as at least one concentration median response value exceeded the efficacy cutoff (ACC). The applied methodology inferred that a positive (toxic) hit call was not derived based on a calculated AC50 value but rather from the curve characteristics, particularly the signal measured above the noise levels and relative to controls. Thus, generated toxicity tables with active hits were further filtered for false positives resulting from cytotoxicity according to two approaches: (1) strict cytotoxicity burst filtering developed previously^53^ (the resulting data set is referred to as +CTB) and (2) a milder filtering procedure removing only the most extreme false positives (the resulting data set is referred to as −CTB). Since tcpl pipeline is semiautomated and data is fitted without manual inspection, modeling outcomes could be artifacts of the curve-fitting workflow. To address that issue, cytotoxicity filtering was followed by a quality evaluation based on caution flags (assigned using measurement and processing meta-data) on the curve-fitting and quantitative uncertainty associated with the curve-fitting. The applied filtering was a modified version of the preprocessing described by Paul Friedman et al.^54^ In addition to the filtering, the data for endpoints covering the same intended biological targets were concatenated. Thus, generated toxicity data were not equally distributed across different endpoints. The number of samples (chemicals) available per endpoint varied from less than 100 to more than 8k. Similarly, the abundance of active hit calls, i.e., toxic cases, for some endpoints was less than 1%, while others reached more than 50% of the available samples. Only endpoints with more than 500 chemicals and more than 0.1% active hits per endpoint were used for modeling. The final data sets (Figures SF2 and SF3) covered 505 (−CTB) and 474 (+CTB) unique endpoints (395 were target-specific, the rest related to viability). In addition to the custom invitroDB data processing and filtering strategy described above and used for model development, a precurated version of the invitroDB from the National Toxicology Programme of the U.S. Department of Health and Human Services (NICEATM) was obtained from the Integrated Chemical Environment (ICE) toolbox (https://ice.ntp.niehs.nih.gov/Tools^55^) and used as a supplementary data set for validation and environmental application of MLinvitroTox. While the ICE data set was significantly reduced in sample size compared to the source data, the data were annotated with the mechanistic target, which can be interpreted as the intended biological effect of each endpoint, such as oxidative stress response or cytotoxicity. More information about the toxicity data and processing can be found in SI (Section Data Processing).

### Structural Data

Structural data for the modeling was obtained from the U.S. EPA’s CompTox Chemicals Dashboard (downloaded as DSSTox_v2000_full.zip via FTP from https://gaftp.epa.gov/). The .sdf file containing structural, chemical, and metadata for approximately 800k chemicals (as of 02.05.2019) was filtered according to the DTXSID (universal compound identifier in the invitroDB and DSSTox), resulting in a list of 10,201 entries. Although the availability of chemical data sets in the public domain, including CompTox, has skyrocketed in recent years, the data contained in such databases is often partially erroneous. The implemented structure cleanup strategy covered the removal of structures that cannot be appropriately handled by conventional cheminformatics techniques (e.g., inorganic and organometallic compounds, counterions, salts, and mixtures) followed by structure standardization and validation (removal of H, ring aromatization, normalization of specific chemotypes, curation of tautomeric forms, and the deletion of duplicates). The code for the cleanup of structures is available in GitLab (https://renkulab.io/gitlab/kasia.arturi/generating-fingerprints.git). Postcuration, the list of chemicals available for modeling decreased to 8k.

Since structures can not be used directly for modeling, they were translated into ML-suitable input: sets of structural and topological molecular fingerprints. Although MACCS and PubChem fingerprints are most commonly used in computational toxicology,^56^ there is little scientific evidence that these fingerprints yield optimal outcomes. For the development of MLinvitroTox, 23 types of fingerprints (Table ST1) were tested and evaluated for predicting activity endpoints covered in invitroDB. The following fingerprints were computed: (1) CDK (Chemistry Development Kit)^57^ via CDK Nodes for KNIME, (2) RDkit (research development kit)^58^ via RDKit Nodes for KNIME, (3) OpenBabel fingerprints^59^ via Pybel,^60^ (4) ToxPrint (https://toxprint.org/) via ChemoTyper (https://chemotyper.org/),^61^ and (5) SIRIUS/CSI:FingerID fingerprints^14,15,62−65^ (referred here simply as SIRIUS fingerprints) via PaDEL^66^ and OpenBabel.^59^ While tools 1–3 generate standard structural or topological molecular fingerprints, ToxPrints, and SIRIUS fingerprints are unique implementations. ToxPrints are a publicly available set of structural keys targeting chemical chemotypes relevant for toxicity according to databases and regulatory inventories.^54,61,61,67−70^ SIRIUS fingerprints are a compilation of structural fingerprints (MACCS, PubChem, OpenBabel, extended connectivity [ECFP], Klekota Roth, custom-made SMARTS, and ring systems). While the 23 types of fingerprints were used for understanding what input is optimal for predicting different types of toxic outcomes, for practical purposes of obtaining molecular fingerprints from fragmentation spectra, SIRIUS/CSI:FingerID fingerprint was the focus of the current work. The code for generating SIRIUS fingerprints is available in GitLab (https://renkulab.io/gitlab/kasia.arturi/generating-fingerprints.git).

### Machine Learning and Data Mining

#### Approach

Machine learning was applied to train binary classifiers for single invitroDB toxic activity endpoints based on molecular fingerprints of structures. The ML workflow utilized in this study, along with the underlying logic and applied parameters, is shown in Figure SF4. The toxicity data files postcuration (binary hit calls, hitc = 1 for toxic and hitc = 0 for nontoxic outcomes for 474 and 505 endpoints in + CBT and −CBT, respectively) were combined with 23 molecular fingerprint types (Table ST1), resulting in more than 20k files for MLinvitroTox development and optimization. Each file was preprocessed (removal of features [in ML context features are variables used for training of the model, i.e., molecular fingerprint bits corresponding to specific substructures], with variability [<5%] and high intercorrelation [>95%]) significantly reducing the number of ML variables to an average of 200–400 per endpoint. The binary classification was chosen in this work to infer toxic behavior over a quantitative regression predicting AC50 or ACC based on the research goals, i.e., prioritization of unidentified signals for further investigation rather than quantification of toxic effects. Although “the dose makes the poison” (Paracelsus), invitroDB chemicals were tested in the same concentration range (0.1 to 100 μM^71^), so a direct comparison is conceivable. Furthermore, chemicals with LC50 values greater than 100 mg/L (equivalent to 200 μM for a compound with a molecular weight of 500 Da) are considered practically nontoxic in aquatic toxicology.^72^ Despite being less commonly employed for toxicity prediction, recent studies have demonstrated that classification can be an accurate and valuable tool for assessing toxicity.^50^ In addition to investigating the impact of molecular fingerprint type and cytotoxicity filtering on toxicity modeling, we also methodically varied the model type as well as oversampling and resampling strategies.

#### Modeling Details

In the initial modeling phase, the Caret package^73^ was employed in R to develop classifiers using 20 different models (method = CSimca, RRF, AdaBoost, bayesglm, deepboost, gaussprRadial, gbm, glmnet, pcaNNet, regLogistic, rf, svmPoly, svmRadialCost, kernelpls, kknn, avNNet, nnet, glmStepAIC, AdaBag, xgbTree) for predicting 130 molecular toxicity endpoints from the −CTB data set (mild cytotoxicity filtering). To account for sparse (low number of samples) and imbalanced (low availability of positive [toxic] training examples) endpoints, five oversampling (none, down, ROSE,^74^ SMOTE,^75^ up) and three resampling (boot = bootstrapping, LGOCV = leave-group-out cross-validation, and repeatedcv = repeated random 10-fold cross-validation) strategies were tested. For each combination of endpoint, model, fingerprint, oversampling, and resampling, a separate model was optimized, including an automated hyperparameter tuning with random grid searching (tune length = 10 based on default values for each model parameters) nested within the validation procedure (resamp parameter above), and fully independent testing on new samples (80/20 split for training/testing partitions). Test sets for each partition were withdrawn from the data prior to oversampling. Oversampling was nested within resampling. Cross-validation of each model was nested within hyperparameter tuning with random grid searching. The computing was performed on Piz Daint, a supercomputer (Cray XC40/XC50, XC40 compute nodes Intel Xeon E5–2695 v4 @ 2.10 GHz [2 × 18 cores, 64/128 GB RAM] 1813 Nodes) from the Swiss Super Computing Center (CSCS) in Lugano (Switzerland). Shell scripts were set up to automatically run the modeling in small batches on multicore (mc) multithread (2–8 CPUs per node) on 10–50 nodes. The total processing time amounted to nearly 20,000 node hours. As each fingerprint/endpoint combination required at least 300 models (20 model types × 5 oversampling strategies × 3 resampling strategies) to be trained (excluding cross-validation and hyperparameter tuning), training classifiers for all combinations of endpoints (−CTB and +CTB) and molecular fingerprints was deemed unfeasible due to computational limitations. Instead, the collected data was used as a representative sample to understand the modeling requirements (relevant model types, optimal oversampling, and resampling strategies) for invitroDB data. A custom KNIME workflow was subsequently built based on autoML^76^ for fine-tuning, validation, and environmental testing in the second modeling stage. For that purpose, 9 models (naive Bayes [NB, 0.004 < def_prob < 0.1], logistic regression [LG, 0.001 < step_size < 0.1], generalized linear models [GLM, 0 < α < 1, 0 < λ < 1], decision tree [DT, 2 < min_num_rec < 20], random forest [RF, 4 < tree_depth < 20, 10 < node_size < 25, 25 < no_of_trees < 200], gradient boosted trees [GBT, 10 < num_trees > 100], extreme gradient boosted trees [XGB, 1 < max_depth < 10, 0.01 < η < 0.3], neural networks [NN, 1 < num_layers < 5, 5 < num_networks < 50], and deep learning with Keras architecture [DL, 10 < num_neurons < 100, 10 < num_networks < 100]) were trained in parallel with repeatedcv resampling (*k* = 10) and SMOTE oversampling for all endpoint/fingerprint combinations and the winning model was found based on F-measure. In this case, hyperparameter tuning grids were defined manually for each model, as indicated above, and searched randomly for *n* = 50 with early stopping at 20. The computations were executed in KNIME on a Windows Server 2012 (64-bit operating system, Intel(R) Core(T.M.) i7-8700K CPU @ 3.70 GHz with 64 GB RAM). Total processing time amounted to more than 150,000 core hours. The same core principles of testing, oversampling, hyperparameter tuning, and cross-validation were applied in all modeling phases.

#### Evaluation

The modeling resulted in more than 500k data points, i.e., metrics for the performance of the models for each combination of endpoint, cytotoxicity (+CTB and −CTB, respectively), molecular fingerprint, model, oversampling, and resampling. The results for different models, each representing a particular algorithm, were summarized (averaged) with model classes: tree (simple tree models), boost-tree (boosted tree models), xboost-tree (extreme gradient boosted tree models), linear (linear regression models), simca (simca classification algorithm), knn (knn classification algorithm), pls (multivariate models), svm (support vector machines models), Gaussian (Gaussian models), and deep (neural networks models). Similarly, the 23 fingerprint types used were concatenated according to the fingerprint type, i.e., CDK, RDKit, ToxPrint, and SIRIUS. The large volume of the generated modeling results was used to infer a general set of rules guiding the modeling of invitroDB data, including the sparse and imbalanced end endpoints often omitted from modeling but included here as part of the data-mining-driven approach. The results were reported according to the guidelines for reporting QSARs.^77^ The models were evaluated based on F-measure (also known as F1 score = 2 × precision × sensitivity/[precision + sensitivity]), which focuses on maximizing the detection of true toxic cases (maximizing true positives) instead of optimizing the overall model performance via, e.g., AUC (area under the ROC curve [receiver operating characteristic curve]). The plots with modeling results are based on sensitivity, precision, and balanced accuracy (1/2·[TP/[TP+FN]+TN/[TN+FP]). While sensitivity (TP/[TP+FN], TP-true positives, FN-false negatives) indicates the rates of true toxic cases detected, precision (TP/[TP+FP], FP-false positives) shows the rates of toxic predictions being true. The combined optimized models are termed MLinvitroTox, a toolbox taking molecular fingerprints as input and outputting toxicity fingerprints, where each bit represents a particular toxic activity behavior.

### Validation with MassBank MS2 Spectra

To verify MLinvitroTox with real-life spectral data, the models were applied on the open-source spectral library MassBank (https://MassBank.eu/MassBank/, release version 2021.12) compiled for identifying small molecules of relevance in metabolomics and exposomics. The MassBank database was filtered from the available 85k MS2 spectra to 40k [M + H]^+^ high-resolution spectra obtained from ESI-Q-TOF and ESI-ITFT instruments. Low-resolution spectra were excluded. Molecular fingerprints for each compound with viable spectra were predicted by SIRIUS v. 5.5.7 with standard settings and 10 ppm mass accuracy. Each spectrum was processed separately, i.e., spectra for the same compound, but different collision energies were treated as separate instances. Per each processed spectrum, a maximum of 10 fingerprints was allowed. As shown by Peets et al.,^48^ SIRIUS fingerprints calculated from the same MS2 are similar, even when an incorrect molecular formula is assigned to the spectrum. Multiple spectra per compound and fingerprints per spectrum resulted in synthetic replicates of fingerprints for each compound (range 1–67, average 8). For each processed spectrum, SIRIUS produced 3878 (pos) bits posterior Platt probabilities, i.e., the probability that a molecular property is present, that were converted to binary SIRIUS molecular fingerprints with a 0.5 threshold (≤0.50 = 0, >0.50 = 1). The following fingerprint types from SIRIUS were used: OpenBabel FP3, OpenBabel FP4, MACCS, PubChem, Klekotha-Roth, custom SMARTS, and ring systems. ECFP fingerprints were omitted as the publicly available cheminformatic packages can not easily compute them. The final number of fingerprint bits available to MLinvitroTox was 2363 (positive mode). In addition, true molecular fingerprints were generated for each MassBank compound from chemical structures via Padel^66^ and Pybel^59,60^ cheminformatic packages and compared to the predicted fingerprints to assess the accuracy of SIRIUS predictions. The generated sets of predicted and true molecular fingerprints for MassBank compounds were used as input to MLinvitroTox to predict the corresponding toxicity fingerprints that were subsequently compared to invitroDB records for performance validation. MLinvitroTox was retrained with optimal configuration (xboost model, 5-fold cross-validation with nested 20-step hyperparameter tuning based on a random grid search, SMOTE oversampling) on a combination of invitroDB and ICE data excluding all MassBank compounds. The inclusion of ICE data enabled grouping of endpoints based on their shared mechanistic target, i.e., the intended biological effect. Although less than half of the existing mechanistic targets are represented in MassBank (23 out of 67), it is one of the most comprehensive currently available open-source MS2 compilations. Each of the retrained models was internally validated (cross-validation with nested hyperparameter tuning on a random grid search) and tested on an “independent” (pulled out of the full data set prior to any modeling) during the classifier training/validation/testing. The final hit call (toxicity) per compound was established by voting from synthetic replicates. Although all available invitroDB endpoints for various mechanistic targets were trained in MLinvitroTox, only endpoints that demonstrated testing sensitivity and precision greater than 0.65 during ML model training were permitted to contribute, i.e., vote on the outcome. The model performance for each mechanistic target was averaged across its endpoints.

### Application to Environmental Water Samples

#### Samples

MLinvitroTox functionality and performance were tested on environmental real-life HRMS/MS raw data from samples collected in 2014 at three wastewater treatment plants (Birmensdorf, Muri, and Reinach) discharging to small streams in the Swiss Plateau, as well as upstream and downstream of the plants, as described in the work of Neale et al.^51^ In short, the samples were prepared using online solid phase extraction (SPE) and analyzed with reverse-phase liquid chromatography high-resolution tandem mass spectrometry (LC-HR-MS/MS, Q-Exactive Plus, Thermo Fisher Scientific). HRMS/MS analysis was combined with bioassays to screen the samples for specific toxic effects and determine which chemicals from a list of 400 common target compounds could be responsible for the measured effects. Initially, only target data processing was performed in TraceFinder. In this study, raw data files were processed using an NTS workflow in MS-DIAL to obtain MS1 feature lists and representative MS2 spectral records of the measured signals. The representative MS2 spectra were exported as mgf files and served as input for SIRIUS/CSI:FingerID to generate corresponding molecular fingerprints, subsequently used to predict the signals’ relevant toxic activities via MLinvitroTox. The overall aim was to (1) confirm with MLinvitroTox the outcomes of experimentally obtained target analysis results and (2) explain the missing (not covered by the target analysis) mixture toxicity caused by unidentified NTS signals.

#### MS-DIAL Processing

The HRMS/MS data were initially recorded in data independent analysis (DIA) positive and negative electrospray (ESI) mode as raw files, subsequently converted to abf format (https://www.reifycs.com/AbfConverter/). In the current study, the data were reanalyzed using NTS workflow (peak-detection, alignment, and gap filling) in MS-DIAL^78^ with a mass list (target compounds) and reference spectral database (MassBank spectral database, https://MassBank.eu/MassBank/, release version 2021.12, msp format containing 90,190 unique spectra for 15,075 compounds). MS-DIAL settings recommended in the literature^78^ were optimized based on the target compounds: (a) Data collection: retention time (RT) begin [3 min], RT end [28 min]; mass range begin [100 Da], mass range end [1000 Da] (both MS1 and MS2), MS1 tolerance [0.01 Da], MS2 tolerance [0.05 Da]; (b) Peak detection: smoothing level [4 scans], minimum peak height [30,000 and 10,000 amplitude in positive and negative mode, respectively], minimum peak width [12 scans], mass slice width [0.1 Da]; (c) MS2Dec: sigma [0.1], MS2 abundance cutoff [0]; (d) Identification: accurate mass tolerance (MS1) [0.001 Da], accurate mass tolerance (MS2) [0.005 Da], RT tolerance [0.5 min], identification score cutoff: 85%. All available adducts definitions were used. The features were aligned across different samples (mass accuracy [0.001 Da], RT tolerance [0.5 min]) and filtered (with the removal of features activated) based on blank (sample/blank ratio 5-fold). SWATH-MS experiment file with the following *m*/*z* ranges was extracted from the raw data and provided during MS-DIAL setup: SCAN 100–1000 *m*/*z*; SWATH: 95–180, 170–255, 245–330, 320–405, 395–1005. Positive and negative mode measurements were processed separately.

#### Feature Processing

The deconvoluted representative spectra for the detected MS1 features (46k features: 18k [neg] and 28k in [pos]) were exported as mgf, imported to SIRIUS, and processed for positive and negative modes together with the same parameters as in the validation with MassBank spectra. Due to the lack of feature componentization in MS-DIAL, replicate MS1 (and MS2) were present for some features, requiring additional filtering. To address this, the MS1 peak lists (measured areas, peak shape information, and signal-to-noise levels) were processed through several steps to refine and consolidate the data. The features were componentized into molecular ions [M + H]^+^ and [M – H]^−^ (mass accuracy [5 ppm] and RT tolerance [0.5 min]), and adducts were removed. Features were then filtered based on their identification status: targets with reference material confirming the structure (from mass lists based on mass and RT, confidence level 1 according to Schymanski et al.^19^), probable candidates (spectral ID based on 85% match with MassBank spectral database, confidence level 2a), and tentative identifications (a partial match with MassBank spectral database, confidence level 3). Quality filters were then applied to any remaining unidentified signals, with only those exhibiting a Gaussian peak shape > 0.8 and signal-to-noise ratio > 10 being retained. This process prioritized 10k unique MS1 signals that were subsequently used for filtering the SIRIUS results computed for all MS2 spectra.

#### Processing in SIRIUS

All exported MS2 spectra were used as input to SIRIUS to retain the information from the synthetic replicates. From 46k deconvoluted MS2 exported as mgf, 42k met the quality requirements and were imported successfully to SIRIUS as ms. From 42k spectra, 26k had an [M + H]^+^/[M – H]^−^ MS1 precursor < 600 Da. Due to the significant increase in processing time associated with larger molecular weights of the precursor and the focus on small molecules in this study, a reasonable limit of 600 Da was established to avoid excessive computational demands. For 22k spectra, 150k molecular fingerprints were computed. 54k of the 150k generated molecular fingerprints belonged to 9.5k out of the 10k prioritized MS1 features and were used as input to MLinvitroTox models. SIRIUS produced 3878 (pos) and 4072 (neg) bits posterior Platt probabilities for each processed spectrum. 2363 and 2483 bits in the positive and negative modes, respectively, were used in MLinvitroTox. In parallel to the computation of predicted molecular fingerprints for NTS signals via SIRIUS, true molecular fingerprints for each target compound were computed as described above and used as input to MLinvitroTox.

#### Processing in MLinvitroTox

In the current work, we focused on 3 mechanistic targetscovered in invitroDB out of the 13 effects from the original study, namely activation of the aryl hydrocarbon receptor (AhR), activation of the androgen receptor (AR), and oxidative stress response (OSR). MLinvitroTox was retrained with optimal configuration (xboost model, 5-fold cross-validation with nested 20-step hyperparameter tuning based on a random grid search, SMOTE oversampling) on a combination of invitroDB (−CTB) and ICE data excluding all MassBank compounds and the target list compounds. Overall, 20 unique endpoints and 5 endpoint concatenations from invitroDB (AR: 18, AhR: 5, and OSR: 2) were used as distinct toxicity instances representing the AR, AhR, and OSR effects. Only endpoints with testing sensitivity and precision >0.65 (during ML model training) were included in the analysis. Since a different set of fingerprint bits are generated by SIRIUS for positive and negative molecular ions (corresponding to separate models trained for predicting the bits by SIRIUS/CSI:FingerID), unique MLinvitroTox models had to be trained and tested for [M + H]^+^ and [M – H]^−^ per endpoint. The final toxic activity hit call (hitc) per signal for each mechanistic target was determined based on the majority votes from hundreds of synthetic replicates created by (1) the presence of multiple spectra per feature (features are not componentized in MS-DIAL), (2) (max.) 10 fingerprints generated per spectrum, and (3) numerous endpoints per mechanistic target. The voting process was implemented to enhance the prediction robustness by incorporating hundreds of data points. For example, if 5 spectra were detected for a compound, 10 fingerprints were generated per spectrum, and 6 endpoints represented the target effect, the toxicity hit was called based on 300 inputs.

#### Global Feature Importance

The MLinvitroTox models developed for AR, AhR, and OSR were analyzed using global feature importance^79^ methodology to extract structural moieties responsible for the predicted toxic effect. In this approach, a set of surrogate random forests was trained on the same features as the xboost-trees, generating an interpretable model that approximated the overall feature contributions of the original black box from the xboost-tree. The structural bits with the highest importance were extracted as SMARTS (Smiles Arbitrary Target Specification) strings and interpreted visually via SMARTS.plus package.^80^ For each studied effect, a top 10 feature list was generated based on the combination of recurrence (across different endpoints corresponding to the same mechanistic target) of a feature with its value of global importance.

## Results and Discussion

### Mining invitroDB

**Figure 2:**
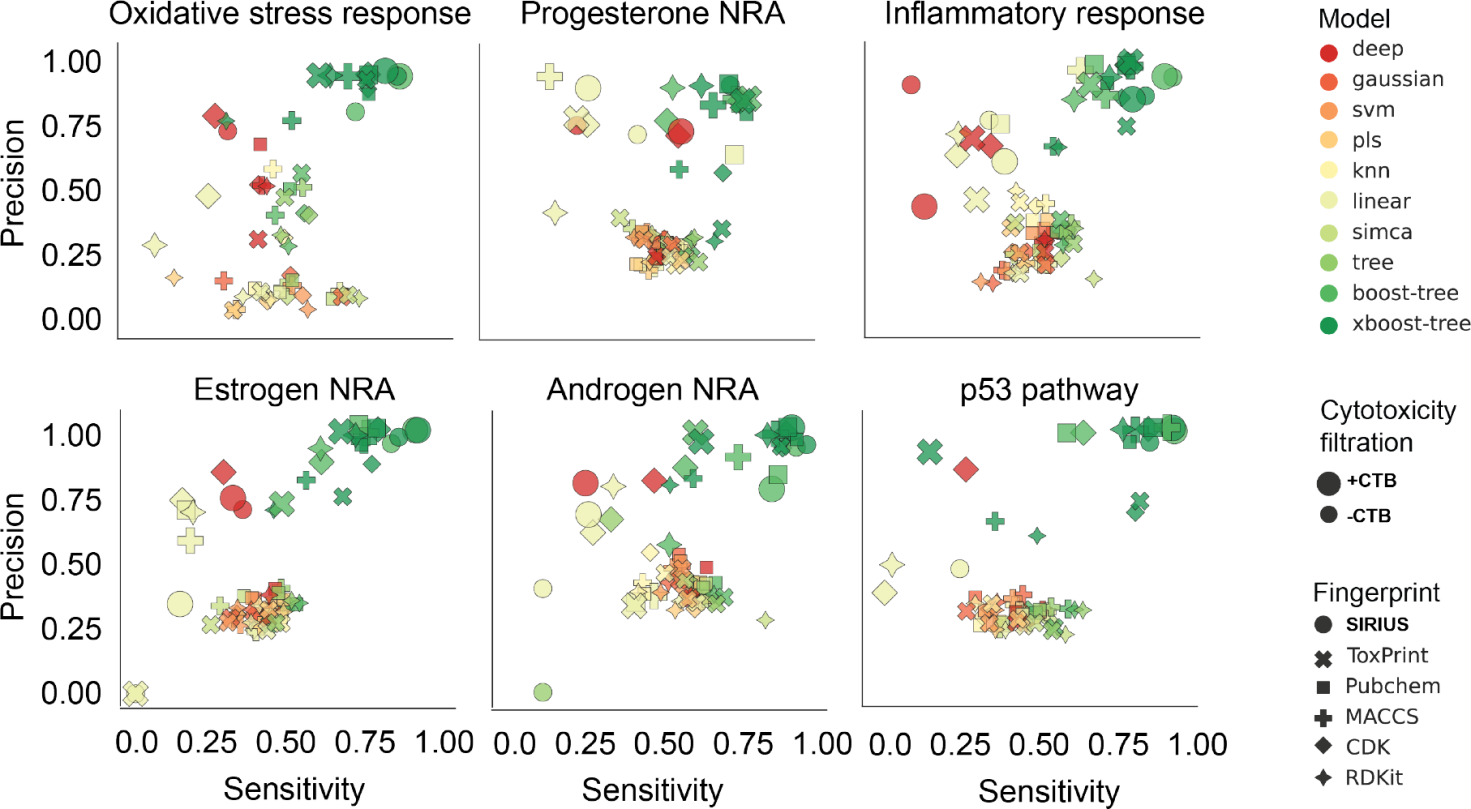
Precision vs sensitivity plots showing MLinvitroTox performance for specific mechanistic targets: endocrine-relatednuclear receptor activation(NRA), oxidative stress and inflammatory responses, and the p53 pathway, often associated with carcinogenicity. Each point on the plots represents the average (across relevant endpoints) model metrics for a specific combination of parameters (model, data set [cytotoxicity], molecular fingerprint, oversampling, and resampling). Figure SF11 shows the corresponding results for all modeled mechanistic targets.

The goal of modeling invitroDB was to determine which of the 395 target-specific and over 100 cytotoxicity-related invitroDB endpoints (derived from data cleanup and processing) can be successfully predicted by ML. The results confirmed that predicting toxic activity from the structure is possible for the majority of endpoints and mechanistic targets with optimized input and processing (Figures SF5–SF8). The combination of xboost-trees and SIRIUS fingerprints emerged as the most successful algorithm and fingerprints, respectively, based on our analysis (Figure SF9). This combination achieved the highest rates of sensitivity (the proportion of toxic cases correctly detected) and precision (the proportion of true positive predictions) for the majority of single endpoints (Figure SF10), as well as for endpoints grouped by their biologically interpretable mechanistic targets (Figures 2 and SF11). xboost-tree models are a special case of decision trees with random input features and combining the outputs from the resulting classifiers for the final decision through a democratic voting process (boosting). In addition, the consecutive models’ parameters are adjusted based on feedback from previous classifiers.^81^ Thanks to their speed and efficiency, xboost-tree models are behind several cutting-edge science^48^ and industrial applications. The success of SIRIUS fingerprints in modeling invitroDB data confirmed that, in theory, the prediction of toxicity based on fingerprints generated from MS2 should be possible. Deep learning methods performed well in precision but resulted in poor sensitivity, most likely due to insufficient data. While certain endpoints contained up to 8k training examples (chemicals), the majority averaged 1–3k. The obtained results (Figure SF6) also underline the importance of proper sparsity and imbalance handling in predicting toxicity, as recently pointed out by Kim et al.^82^ While several oversampling approaches resulted in acceptable outcomes, doing nothing (oversampling none) resulted in the least accurate models. Oversampling creating jittered synthetic minority class samples (SMOTE) was the most successful. The optimal ML configuration (xboost-tree model, SIRIUS fingerprint, SMOTE oversampling, and repeatedcv resampling) resulted not only in high detection rates of toxic cases (sensitivity) but overall model performance (accuracy and precision) as shown in Figure SF12 with a consistent performance across the endpoints belonging to the same mechanistic target. In addition, the obtained model metrics have shown no strong correlation between the number of chemicals available for training or the positive hit rates (Figure SF13), indicating that successful models were computed not only for endpoints with a lot of data available but also the sparse and imbalanced data sets thanks to the applied optimization and oversampling strategies.

The effect of cytotoxicity filtering on the modeling outcomes was not as easily interpretable as the other studied parameters (Figure SF8). A closer analysis of the results for single endpoints and across mechanistic targets revealed a more complex relationship between cytotoxicity filtering and model metrics, primarily dependent on the data sample size and toxic hit rates. For endpoints with a large sample size (8k) and a significant number of toxic examples (percentage of positive hit calls larger than 10%), cytotoxicity filtering had, as expected, an overwhelmingly positive effect. In this case, the removal of false positive chemicals diluting the patterns responsible for toxicity out-weighted the removal of a few true positives. On the other hand, for imbalanced (percentage of positive hit calls less than 1%) and sparse data sets (1k), removing even a few true cases had a more distinct negative effect on the model metrics. In summary, cytotoxicity burst filtering should be performed on an endpoint basis. Alternative strategies, such as baseline toxicity filtering,^83,84^ have shown promise for addressing cytotoxicity in *in vitro* data and will be considered in the future versions of MLinvitroTox.

### MassBank Validation

**Figure 3:**
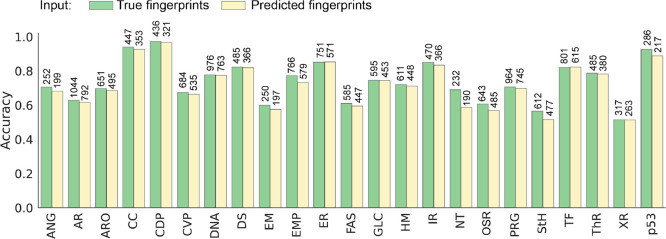
Comparison of MLinvitroTox performance (average balanced accuracy across relevant endpoints) for classifying MassBank compounds as toxic/nontoxic according to 23 mechanistic targets with structures (True, based on 1.5k MassBank structures with invitroDB records) and MS2 (Pred, based on 1k MassBank spectra with invitroDB records for which molecular fingerprints could be generated in SIRIUS) as input. The exact number of chemicals available for validation per mechanistic target is provided above the columns. **ANG**, Angiogenic process; **AR**, Androgen receptor; **ARO**, Aromatase; **CC**, Cell cycle; **CDP**, Cell death; **CVP**, Cell viability; **DNA**, DNA damage; **DS**, Developmental signaling; **EM**, Extracellular matrix; **EMP**, Energy metabolism; **ER**, Estrogen receptor; **FAS**, Fatty acid signaling; **GLC**, Glucocorticoid receptor; **HM**, Histone modification; **IR**, Inflammatory response; **MOA**, Monoamine oxidase; **NT**, Neurotransmission; **OSR**, Oxidative stress response; **PRG**, Progesterone receptor; **StH**, Steroid receptor; **TF**, Transcription factors; **ThR**, Thyroid receptor; **XM**, Xenobiotic response; **p53**, p53 pathway.

Although toxicity fingerprints were successfully predicted by MLinvitroTox from structures, predicting them from molecular fingerprints based on MS2 remained to be proven. For that purpose, MLinvitroTox was externally validated on MassBank spectra (https://MassBank.eu/MassBank/, release version 2021.12). In short, for 40k MS2 spectra (corresponding to 4k unique compounds), 20k fingerprints (corresponding to 2.5k unique compounds) were generated in SIRIUS. The spectra quality of the missing 1.5k compounds was insufficient (low resolution, too few fragments, noisy) to generate molecular fingerprints via SIRIUS. Out of the 2.5k compounds, 1k had existing records in invitroDB, enabling a direct comparison of MLinvitroTox predictions with experimental outcomes. In addition, for the 2.5k compounds, the quality of the predicted SIRIUS fingerprints was compared to the true fingerprints generated from structures. Although a certain divergence between the predicted and true fingerprints was observed (Figure SF14), the overall average accuracy for the prediction of the presence and absence of different substructures was satisfactory (balanced accuracy 98.5%, sensitivity 90.8%, Tanimoto coefficient 0.89) varying depending on the combination of quality of the input MS2 spectra and the intrinsic accuracy of SIRIUS predictors. The imperfect input for validation mimicked a real-life application of MLinvtroTox, where neither the structures nor the molecular fingerprints for unidentified MS1 features are available. As shown in Figure 3, MLinvitroTox exhibited a robust balanced accuracy in predicting toxicity from both structures (0.75), as well as MS2 (0.74) for the MassBank compounds despite the flawed input. It is worth reminding that prior to model development, feature filtering reduced the input from 2.5k to a more manageable 200–400 variables per endpoint. While the outlined DTXSIDs counts represented unique compounds, the number of independent votes by synthetic replicates (multiple spectra per compound and up to 10 fingerprints per spectrum from SIRIUS) per endpoint was typically multiple times higher, providing more confidence in the outcome. It should be mentioned that SIRIUS was trained on a data set of 14k compounds, which included 2k compounds from MassBank. Of the 1k MassBank compounds that had invitroDB records and were used to validate MLinvitroTox, 596 compounds were also present in the training set of SIRIUS. Therefore, molecular fingerprints’ predictions for this subset were potentially more accurate than what could be expected from a truly independent data set. After excluding the subset common for SIRIUS and MassBank from the MLinvitroTox validation, we found that the average accuracy of the models did not show a significant decrease (Figure SF15).

### Environmental Application

Although predicting toxicity fingerprints from molecular fingerprints computed based on relatively pure spectra, such as those present in the MassBank spectral library, was successful, the ability of MLinvitroTox to do the same using real-life environmental HRMS/MS data remained to be demonstrated. Samples of wastewater and surface water collected and analyzed by Neale et al.^51^ were used to test MLinvitroTox in the environmental context. The original study combined target HRMS/MS analysis of 400 common pollutants with 13 bioassays to explain mixture toxicity. According to the results, for most of the studied toxic outcomes, only a fraction of the measured effect could be explained by the detected targets, indicating the presence of additional/more potent pollutants in the samples. In the current study, MLinvitroTox was applied to confirm the target analysis and find unidentified features potentially responsible for the missing mixture toxicity in AR (androgen receptor), AhR (aryl hydrocarbon receptor), and OSR (oxidative stress response). From the 46k MS1 features detected via the NTS processing workflow in MS-DIAL, 9.5k were prioritized based on ID and signal quality and the availability of experimentally predicted SIRIUS fingerprints (54k molecular fingerprints). The distribution of IDs among the prioritized features was as follows: (A) 268 targets (235 [pos] and 33 [neg]) with confidence level 1 according to Schymanski et al.^19^ (confirmed with reference standard); (B) 109 signals automatically matched to MassBank library records providing a confidence level 2a (probable structure) identification; (C) 2k features with tentative identification (MS1 match, partial MS2 match, confidence level 3); and (D) 7k signals without any identification.

**Figure 4:**
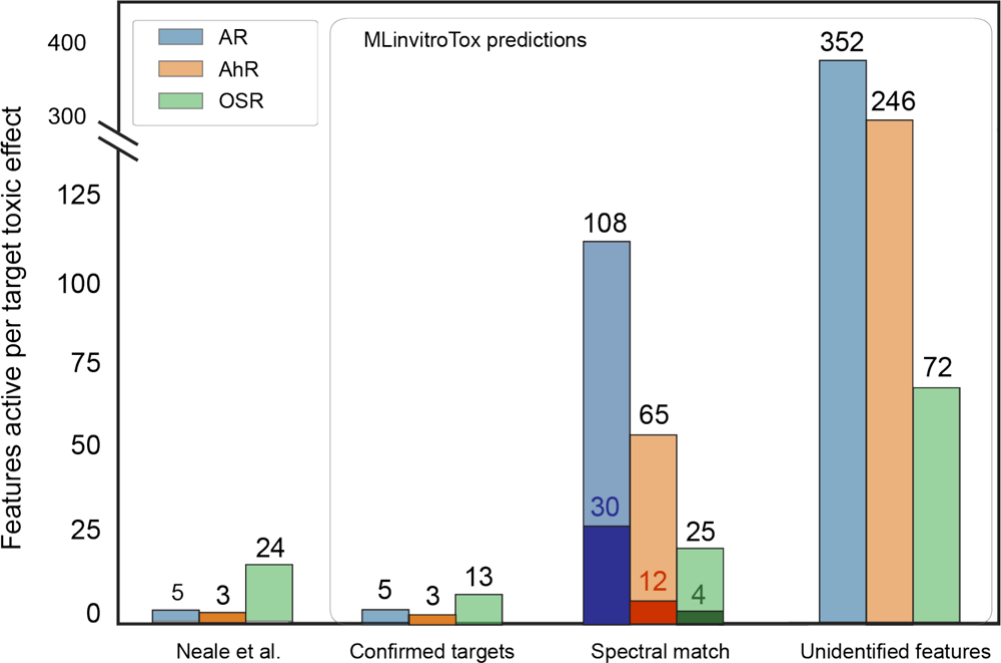
A summary of MLinvitroTox performance for predicting AR, AhR, and OSR for HRMS/MS features from environmental samples. MLinvitroTox predictions were consistent with the experimental results obtained by Neale et al.^51^ for Confirmed targets. In addition, 783 NTS signals were linked to potential toxicity in AR, AhR, or OSR, divided on the figure into Spectral match (automatic MassBank reference database match by MS-DIAL) and Unidentified features. The dark portion of the bars represents the portion of compounds for which the predicted toxicity matched with invitroDB records.

The results for the MLinvitroTox target analysis are summarized in Figure 4 and Figure SF16. For AR and AhR, MLinvitroTox confirmed the toxicity of all relevant targets (explaining less than 1 and 30% of the total mixture toxicity in the original study) using the true and predicted molecular fingerprints as input. For OSR, the toxicity of 13 out of 24 compounds (Table ST2) was predicted correctly, including 7 out of 9 of the most potent ones. For one of the compounds (caffeine), the toxicity could not be confirmed via either the true or the predicted fingerprints. Although a reasonably good model performance was obtained for the oxidative stress response mechanistic target (Figure 2), modeling OSR may be more challenging since it is not a molecular initiating event (MIE) like AR or AhR, but a key event (KI). For telmisartan, no molecular fingerprints were generated. Out of the 24 OSR-active compounds from the original analysis, molecular fingerprints were predicted by SIRIUS for 18 (13 in positive and 5 in negative mode). The spectra’s quality was insufficient to generate molecular fingerprints for the remaining 6 OSR-relevant compounds detected in the negative ESI mode. In general, obtaining correct predictions was likely more challenging because the MS2 spectra were obtained in DIA (data-independent analysis mode), for which raw data processing is considered less reliable than DDA (data-dependent mode).

MLinvitroTox was not only able to confirm the majority of chemical/endpoint interactions for targets detected by HRMS/MS but also tag 783 additional NTS features (corresponding to 868 chemical/target interactions) as toxic in AR, AhR, or OSR, including 109 features (Table ST3) with a spectral match (corresponding to 198 chemical/target interactions), thus potentially explaining the missing mixture toxicity (Figure 4). For 30 out of 109 compounds with spectral matches, the predicted toxicity in AR, AhR, and OSR could be confirmed by invitroDB data (corresponding to 46 toxic chemical/endpoint interactions). Nine additional compounds were falsely predicted to be toxic according to invitroDB records, showing a moderate false discovery rate (FP/[TP+FP]) of 23%. With minimal effort, the pool of potentially toxic compounds from the original analysis was increased significantly by applying MLinvitroTox. We suggest mining various online databases for additional toxicity information for the tentatively identified compounds without invitroDB records. A tentative identification via a traditional NTS pipeline is necessary for the unidentified features with potential toxicity. Since the quality of a spectral match can vary (Figures SF17–SF19), following the application of the MLinvitroTox and data mining of the outcomes, an in-depth analysis adding mechanistic context to the results should take place, resulting ultimately in confirmation or rejection of the predictions.

The models developed for AR, AhR, and OSR and tested on the environmental data were further analyzed using the global feature importance (GFI) methodology^79^ to extract structural moieties responsible for the predicted toxic effect. The aim was to explain the modeling mechanistically beyond the typical ML black box outcome. Figure SF20 shows the distribution of the obtained importance values across all input features and the recurrence of bits across the endpoints for each mechanistic target. On average, 200–400 substructures per endpoint were a standard input to the modeling and the global feature importance analysis. Overall, the distribution of the GFI values in the range of 0–1.75 was skewed heavily to the left, indicating a decreasing number of bits (structures) of increasing importance. The number of substructure recurrences (the same structures were tagged as important for one mechanistic target by multiple endpoints) varied between 1 and 13, depending on the effect. The complete list of substructures generated a top 10 ranking for each mechanistic target (Figure SF21). The overall insights were not surprising: (A) Heterogeneity, i.e., the presence of N and O, in particular, seemed to be associated with toxic effects in general; (B) Matching similar structural moieties was significant for the corresponding mechanistic targets in both directions, i.e., agonist (gain) and antagonist (loss), indicating a certain chemical specificity of a particular receptor; (C) AR was activated by heavily substituted aromatics, in particular, phenols; (D) AhR seemed to be activated by aliphatic compounds with nitrogen (agonist) and oxygen (antagonist); (E) OSR seemed to be associated with less substituted aromatics, but it should be kept in mind that OSR is a KE and not MIE, so the predictions are not directly linked to chemical structures. Although fully explaining particular toxicity based on a limited number of substructures is likely too simplistic, gaining mechanistic insights has many possible applications, e.g., translation of the identified toxic substructures into spectral features would enable near real-time (i.e., during HRMS/MS measurements) detection of potentially harmful compounds, as demonstrated previously by Meekel et al.^85^

### Limitations and Applicability

While MLinvitroTox has successfully predicted toxic activities in various molecular endpoints and mechanistic targets based on both structural as well as MS2 data, it is important to note that, like all modeling approaches, it is not without limitations. First and foremost, the predictions represent molecular toxicity events at a cellular level that do not necessarily result in adverse outcomes on the organ or organism level. To connect HTS bioassays with toxicity in aquatic and human organisms, AOPs are being developed. Second, MLinvitroTox is a binary classifier, i.e., it calls a toxic/nontoxic hit on the tested unidentified features but currently lacks a potency element. Predictions from MLinvitroTox are thus only meaningful for toxicants exhibiting undesirable effects in the concentration range tested in invitroDB,^71^ i.e., approximately 0.1 to 100 μM.^72^ Overall, the accuracy of MLinvitroTox predictions is limited by the quality of the provided MS2 spectra. Poor MS2 spectra (noisy with few fragments) are an inadequate basis for generating molecular and toxicity fingerprints. Similarly, since the models were trained and optimized for molecular fingerprints generated from structural data but are applied to fingerprints generated from MS2, their accuracy is limited by the accuracy of SIRIUS fingerprint prediction. Most importantly, MLinvitroTox was developed for prioritization, i.e., it should be used as the basis for further analysis and not final decision-making. Ideally, applying MLinvitroTox and mining the outcomes should be followed by an in-depth analysis adding research context and expert knowledge to the results, ultimately resulting in an analytical confirmation (or rejection) of the identity and toxicity of the prioritized features. An open-source version of the core elements of the current version of MLinvitroTox (v1.0) implemented in KNIME can be obtained from 10.25678/0007QS along with the HRMS/MS data used for the environmental application and supplementary R scripts. To enhance the integration of MLinvitroTox, we are currently developing an automated pipeline EXPECTmine (Mining Toxicity and Mass Spectrometry Data for Linking Exposures to Effects), equipping the hazard-driven prioritization provided by MLinvitroTox with a potency element and incorporating it into the existing NTS framework for tentative identification and quantification for purposes of, e.g., a risk-based early warning system.

## Supporting Information Available

MLinvitroTox principles, data processing, MLinvitroTox development, MassBank validation, and environmental application (PDF)
