# Supplementary material for: Machine Learning-Based Hazard-Driven Prioritization of Features in Nontarget Screening of Environmental High-Resolution Mass Spectrometry Data
Source: Environ Sci Technol. 2023 Jun 6;57(46):18067–79. doi: 10.1021/acs.est.3c00304 (PMC10666537; doi:10.1021/acs.est.3c00304)
Supplement: Supplementary file 1 — es3c00304_si_001.pdf [file es3c00304_si_001.pdf]

# **Supplementary Information:** Machine learning-based hazard-driven prioritization of features in nontarget screening of environmental high-resolution mass spectrometry data

Katarzyna Arturi[1], Juliane Hollender[1,2]

[1] Department of Environmental Chemistry, Swiss Federal Institute of Aquatic Science and Technology (Eawag), Ueberlandstrasse 133, 8600 Dübendorf, Switzerland

[2] Institute of Biogeochemistry and Pollution Dynamics, Eidgenössische Technische Hochschule Zürich (ETH Zurich), Rämistrasse 101, 8092 Zürich, Switzerland

Correspondence to: kasia.arturi@eawag.ch

**Table of Contents:**

S1 MLinvitroTox Principles

S2 Data Processing

S3 MLinvitroTox Development

S4 MassBank Validation

S5 Environmental Application

## List of Figures


SF1 Concept behind the MLinvitroTox. . . . . . . . . . . . . . . . . . . . . . . S3

SF2 Properties of +CTB and -CTB datasets. . . . . . . . . . . . . . . . . . . . . . S6

SF3 Effects of filtration on data volume. . . . . . . . . . . . . . . . . . . . . . S9

SF4 ML workflow in the current study. . . . . . . . . . . . . . . . . . . . . . . S14

SF5 Precision vs. sensitivity for combinations of endpoint/parameters. . . . . . S15

SF6 Kernel density estimation for the initial modeling phase. . . . . . . . . . . S16

SF7 Kernel density estimation for the fine-tuning phase. . . . . . . . . . . . . . S17

SF8 Kernel density estimation for the optimal configuration. . . . . . . . . . . . S18

SF9 Heat map with sensitivity for fingerprint/model combinations. . . . . . . . S19

SF10 Sensitivity of the best model for each endpoint. . . . . . . . . . . . . . . . S19

SF11 Precision vs. sensitivity according to mechanistic target. . . . . . . . . . . S20

SF12 Compilation of modeling outcomes for the optimal ML configuration. . . . S21

SF13 Heat map with the correlation of metrics. . . . . . . . . . . . . . . . . . . S21

SF14 True vs. predicted fingerprints for MassBank compounds. . . . . . . . . . . S22

SF15 MLinvitroTox validation with MassBank. . . . . . . . . . . . . . . . . . . . S23

SF16 MLinvitroTox on targeted environmental data. . . . . . . . . . . . . . . . . S24

SF17 MS2 spectra of features tagged as toxic (1). . . . . . . . . . . . . . . . . . S29

SF18 MS2 spectra of features tagged as toxic (2). . . . . . . . . . . . . . . . . . S30

SF19 MS2 spectra of features tagged as toxic (3). . . . . . . . . . . . . . . . . . S31

SF20 Global feature importance. . . . . . . . . . . . . . . . . . . . . . . . . . . S31

SF21 Toxic structural moieties. . . . . . . . . . . . . . . . . . . . . . . . . . . S32


## List of Tables


ST1 Types of molecular fingerprints used for MLinvitroTox development. . . . . S13

ST2 Correct MLinvitroTox predictions of targets. . . . . . . . . . . . . . . . . . S24

ST3 Probable structures of NTS features. . . . . . . . . . . . . . . . . . . . . . S25

# S1. MLinvitroTox Principles

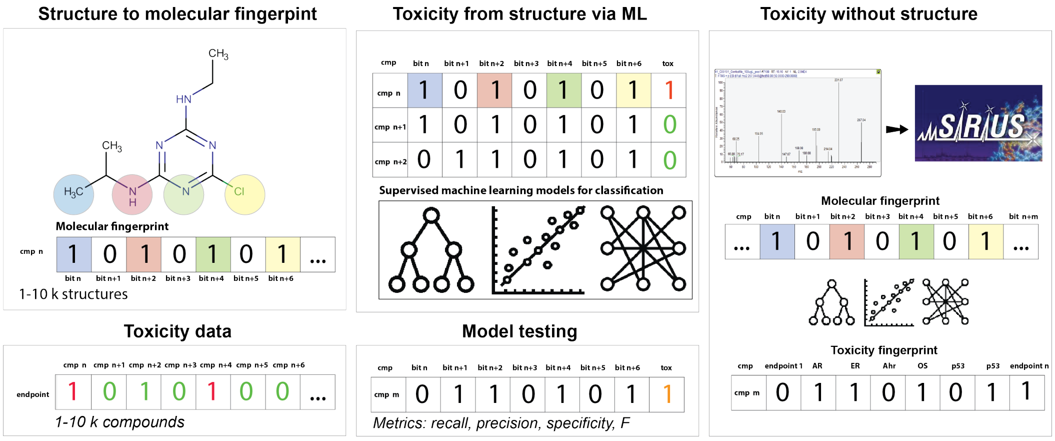


Figure SF1: Concept behind MLinvitroTox, an assembly of machine learning models trained on chemical structures but predicting toxicity from molecular fingerprints generated from MS2 spectra.

Figure SF1 shows the principle of toxicity prediction in MLinvitroTox compared to the traditional approaches. In the latter case, models are trained on molecular fingerprints generated from structures, and in the test phase, the toxicity of known structures for which fingerprints are computed is predicted based on the trained models. In MLinvitroTox, in contrast, the models are also trained on structures. However, in the test phase, the molecular fingerprints for toxicity prediction of unidentified HRMS/MS features are generated via SIRIUS.

# S2. Data processing

**invitroDB.** For toxicity modeling, high-throughput screening (HTS) *in vitro* toxicity data (invitroDBv3.3, `https://doi.org/10.23645/epacomptox.6062623.v6`) from the Environmental Protection Agency (U.S. EPA) was downloaded as MySQL database from `https://www.epa.gov/chemical-research/exploring-toxcast-data-downloadable-data` and installed locally. The invitroDB data includes for each bioassay/chemical pair raw and

processed data outcomes generated through 'tcpl,' chemical and bioassay information, model summaries, and various points of departures (e.g., AC50 [Concentration whereby 50% of the maximal response was achieved, quantitative] and hit call [categorical]). The raw data consists of dose-response curves for chemical/molecular toxicity endpoint combinations generated from 0.1 to 100$\mu$M with dimethyl sulfoxide as a chemical delivery agent. The data was generated by more than 15 commercial and federal government platform sources. Assays were either cell-based or biochemical. The majority of bioassays (>75%) were human cell-based, followed by rat ($\approx$20%) and a minority covered by a wide range of organisms (e.g., bovine, chimpanzee, guinea pig, bovine, mouse, pig, rabbit, zebra-fish, sheep, Chinese hamster). The most common cell lines used included HEK293 (derived from human embryonic kidney cells grown in tissue culture), HEPG2 (human liver cancer cell line), hepatocytes, HeLa (human cancer cell line), and HCT116 (human colon cancer). The binding and enzymatic activity were the most common biological activity types (approx. 40% of the data each), followed by reporter gene activation, signaling, and developmental defects. Some assay endpoints were mapped to genes (1398 unique gene targets). The intended molecular chemical/target interaction was described with the 'Intended target family' covering a large number of biological responses (apoptosis, cell adhesion, cell morphology, growth, DNA binding, various enzymes [esterase, hydrolase, kinase, ligase, lyase, oxidoreductase], and mutagenicity) but with a large focus on nuclear receptors, cell cycle, and cell stress. Overall, invitroDB data spans nearly 800 high-throughput assays and 1473 (105 assays associated with cytotoxicity) molecular toxicity endpoints tested selectively across more than 10k chemicals generating more than 3.72 million toxicity data points available for modeling. The data covers a range of high-level cell responses corresponding to 67 mechanistic targets and more than 300 signaling pathways associated with more than 400 AOPs. invitroDB development (`https://www.epa.gov/sites/default/files/2018-04/documents/toxcastownermanual4252018.pdf`) and its applications[1–10] are described elsewhere.

**tcpl processing.** invitroDB data includes vendor-specific (13 different sources) readouts (level 0, l0) measured both in multiple (mc) as well as single (sc) concentration modes, the normalized and averaged replicates (levels 1-3, l1-l3), dose-response data fitted to constant, hill, and loss-gain models (levels 4-5, l4-l5), as well as flagged data (level 6, l6) and resampled modeled data (level 7, l7). Data l5 were used for modeling. Data l6 and l7 were used for quality filtering and modeling evaluation. Only the multiple concentration dose-response curves (mc) were used for modeling, as sc values do not provide enough information for quality evaluation. The best model was selected based on Akaike Information Criterion (AIC) computed for l5 data fitted to three models (constant, gain-loss, and hill). Several points of departure (POD) were subsequently computed for the winning model: AC10, AC50, ACC, and ACB. AC10 and AC50 (concentration at 10 and 50% of activity, respectively) are equivalent to the EC10 and EC50 in *in-vivo* studies (concentration at 10 and 50% of the effect, respectively). The baseline band (BMAD) was three times the median absolute deviation (3·MAD) overall responses of the two lowest concentrations around the zero effect. While ACB represented the activity concentration at baseline activity, ACC (concentration at the user-defined cutoff value) was computed at 6·BMAD% cutoff. The fitting algorithm required a concentration series with not less than four concentrations, including at least one median (median for concentration replicates) above the baseline (ACB). A dose-response series was assigned an active hit-call label (hitc=1) when either the hill or gain-loss was the winning model, and both the modeled curve fit top (modl_tp) as well as at least one concentration median response value exceeded the efficacy cutoff (ACC). In addition to the quantitative modeling and the final hit calling, the retrieved data included detailed assay information for filtering and concatenation.

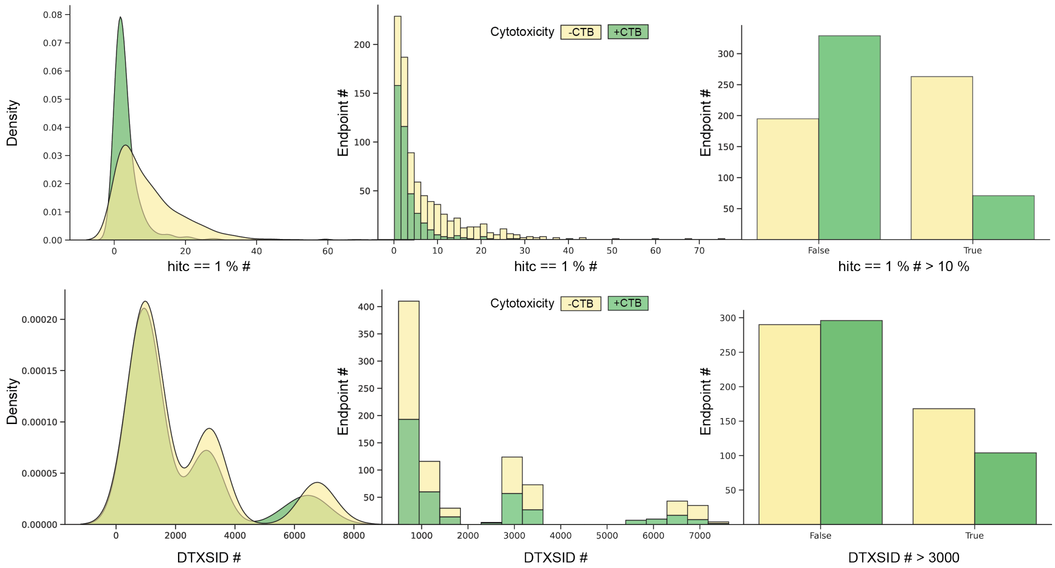


Figure SF2: This figure compares two datasets, one with extensive cytotoxicity filtering (+CTB) and one without (-CTB), with a focus on DTXSID count (bottom) and toxic hit rates (top). The right figures show how cytotoxicity filtering changed the abundance of endpoints with more than 3000 DTXSIDs and positive hit rates (>10%). The threshold of 3000 for the number of chemicals present was used as a boundary between rich and sparse datasets. The figure highlights the impact of filtering steps on the analyzed datasets' characteristics.

**Cytotoxicity filtering** In many cell-based high-throughput screening assays at higher doses, target-specific activation is replaced by cytotoxicity occurring because the chemical starts to disrupt the normal functioning of the cells. In a broader sense, positive calls related to cytotoxicity, adaptive stress responses, or general disruption of molecular machinery are termed cytotoxic burst (CTB). The tcpl package includes methodology, termed the cytotoxicity window approach, for defining chemical-specific CTB estimates to be used as filters for the whole database, even if a chemical in question has no experimental cytotoxicity estimates.[11] In the cytotoxicity window approach, each tested chemical's cytotoxicity point (cyto_pt) is determined simply as the median AC50 with hitc=1 from burst assay endpoints. In addition to cyto_pt, a MAD (median absolute deviation) for AC50 from all endpoints is calculated. Once the burst distribution (cyto_pt point and

MAD) is defined for each chemical, the global burst MAD is determined as the median of the individual MADs. Lower cytotoxic boundary (lower_bnd) is then found as cyto_pt -3·global MAD, and only chemicals with modl_ga_um < lower_bnd (modl_ga_um corresponding to the value of AC50 in the gain direction for the winning model) are kept after the filtering. For chemicals with less than five active hitc, cyto_pt = 3 corresponding to modl_ga_um=1000 $\mu$M was used. More details about the methodology can be found elsewhere.[11]

The cytotoxicity window method reduced the data volume significantly (dataset +CTB), namely from 308k to 68k positive hit calls across all endpoints (Figure SF3 and SF2). Although some of the removed points were undoubtedly false positives, the population of true positives was most likely reduced significantly. According to EPA's recommendations,[12] cytotoxicity filtering should not be used indiscriminately but as a tool for data interpretation and contextualizing the results. The unnecessary removal of toxic cases influences modeling due to the sparsity of the positive training examples. To evaluate the influence of cytotoxicity filtering on the modeling outcomes, a complementary dataset with milder cytotoxicity filtering (dataset -CTB, modl_ga_um < 10·lower_bnd instead of modl_ga_um < lower_bnd) was compiled and analyzed, keeping 271k from the original 308k positive hit calls. Cytotoxicity and the following filtering steps were performed in KNIME (Konstanz Information Miner), an open-source data pipelining platform integrating various modular components for data processing, including cheminformatics, mass spectrometry, data science, machine learning, and data mining through.[13] Besides the built-in KNIME nodes, community-based nodes from CDK (Chemistry Development Kit), RDKit (Research Development Kit), Alvascience, Jchem (ChemAxon), Erlwood, and Vernalis nodes were used.

**Quality filtering.** ToxCast data pipeline is semi-automated, and data is fitted without manual inspection, which can lead to modeling outcomes that are artifacts of the curve-fitting workflow. To address that issue, cytotoxicity filtering was followed by a quality evaluation based on caution flags on the curve-fitting (from level 6) and quantitative uncertainty associated with the curve-fitting (from level 7). The applied filtering was a modified version of the pre-processing described by Paul Friedman et al.[12] The aim was to remove more uncertain data points and increase overall data confidence. Level 6 flagging information denotes curve behavior that may indicate various problematic situations, such as curves based on a single active concentration, AC50 value lower than the lowest concentration screened, borderline activity, efficacy less than 50%, and general indicators of excessive noise and overfitting. In the current dataset, 0-6 flags were associated with each data point, and we chose to remove the bulk of data with 3 or more flags. Level 7 uncertainty information was generated by toxboot R package[14] using smooth, non-parametric bootstrap resampling to define the curve fit's reproducibility. This was performed by 1000 iterations of adding random normally distributed noise to dose-response curves, refitting them, and finally calculating the probability of a positive hit-call given the resampled results. The data was then filtered based on the probability threshold value of 0.5. In addition to level 6 and 7 filtering steps, quality criteria were defined based on level 5 data fit categories, including points with AC50 value below the lowest concentration screened and borderline active points (fit categories 36 and 45, respectively). As the last step, points with no DTXSID [universal compound identifier in the invitroDB and DSSTox] (e.g., polymers), no CASNR (mixtures), and hitc=-1 (not enough data for fitting) were removed.

In the final data processing step, endpoint outcomes were concatenated (aggregated) according to the intended biological target (encompassing the following invitroDB categories: 'biological process target,' 'intended target family,' intended target family sub,'

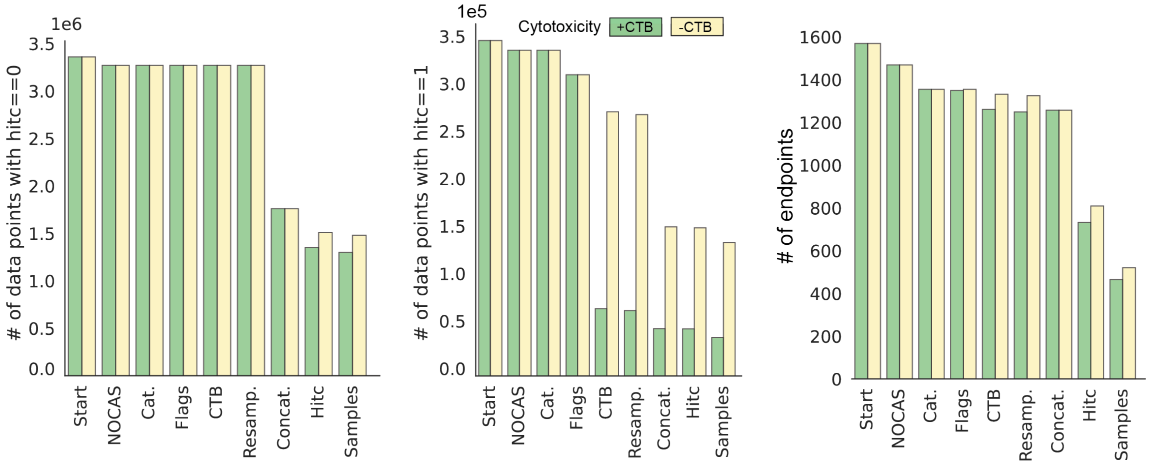


Figure SF3: The effects of each invitroDB toxicity curation step on the available data volume, measured by the number of nontoxic cases retained (hitc=0, left), the number of toxic cases retained (hitc=1, middle), and the number of endpoints (right). The figure provides insights into how each curation step impacts the amount and type of data available for analysis.

'intended target type,' 'intended target type sub,' 'gene symbol,' and 'signal direction') into endpoint classes with a hit call generated by voting. As a result, the number of endpoints decreased, while the number of chemicals, particularly those with positive hit calls, increased. Furthermore, aggregation reduced noise in the data while increasing the confidence in the final hit call, also providing a measure of uncertainty. Only the endpoints with more than 500 chemicals tested and a positive hit rate larger than 0.5% were kept. Figure SF3 shows the effect each step (cytotoxicity, quality, concatenation, etc.) had on the number of endpoints and the data volume, particularly on the hitc=1 population. The final datasets were based on 505 [-CTB] and 474 [+CTB] endpoint classes (395 were target-specific), which, for the sake of simplicity, are here referred to as endpoints. The datasets with strict and mild cytotoxicity filtering, +CTB, -CTB, respectively, contained 816k (+CTB) and 1021k (-CTB) unique chemical/endpoint combinations. As can be seen in Figure SF2, there were three distinct clusters of endpoints with averages of 1.5 k, 3 k, and 7k unique chemicals available for modeling. DTXSID is a unique chemical identifier used across all invitroDB tables to refer to chemicals. The influence of CTB filtering is

seen in all three density regions, most significantly for the last group (7 k). Globally, the average active hit calls fraction decreased from 9.6 to 3.5 between -CTB and +CTB, with the 50th percentile at 6.89 vs. 2.17%, respectively, as the average number of chemicals per endpoint decreased from 2226 to 2038. Although the reduction only represents 8% of all available values, the step had severe consequences for the toxicity information contained, as all removed points were positive hit calls.

**ICE data.** In addition to invitroDB toxicity data, pre-curated invitroDB (termed here invitroDB_ICE or cHTS) data from the Integrated Chemical Environment (ICE) toolbox from the National Toxicology Programme of the U.S. Department of Health and Human Services (NICEATM) was obtained from the Curve Surfer tool available at `https://ice.ntp.niehs.nih.gov/Tools`. The data was retrieved from the EPA invitroDBv3.4 (October 2021), analyzed using the ToxCast Pipeline (tcpl, version 2.0.2), and curated both on the assay and chemical level. Assay curation included the removal of the following assay endpoints (a) channel endpoints (while keeping the corresponding ratios), (b) Tanguay assays of zebrafish, (c) Attagene assays analyzed in the down direction, (d) Background readouts, which resulted in a reduction in the number of dose-response curves from 3.7 million to 620 k. In addition to meeting the tcpl algorithm criteria for an active hit [(i) the best-fit curve model for the concentration series reflects a concentration-response relationship [gain-loss or hill as a winning model], and (ii) the concentration-response curve exceeds the minimum activity cutoff threshold for each assay], for a curve in cHTS to retain the 'active' call it must not: (a) belong to categories 11 (marginal efficacy), and 16 (likely overfitting), (b) have AC50 extrapolated above the tested concentration range, (c) have AC50 extrapolated below the testing concentration range for the gain-loss model, (d) have gain-loss winning model as the winning model for a down-direction assay (i.e., inhibition, antagonism, loss-of-signal), (e) have a single concentration median above the activity cutoff threshold for the gain-loss model. These points were not removed but marked

with 'Flag.omit'. In addition to the curation of assays, chemicals originating from samples with low-quality grades (Q.C. of 'D,' 'F,' 'N.S.,' 'A.C.,' 'F.C.,' 'CC,' or 'B.C.' as annotated in invitroDB), were tagged 'QC-omit.' The tagging reduced the data volume further to approx. 420k dose-response curves. For the complete list of curation filters, please refer to the ICE documentation (`https://ice.ntp.niehs.nih.gov/DATASETDESCRIPTION?section=cHTS`). The strict curation criteria significantly affected the volume of the toxicity data available. While the number of endpoints was retained (reduced from 1364 to 1253), the average number of chemicals available for training fell from over 2k before the curation to 331, including 127 active hits calls per assay endpoint. Overall, only 219, 83, and 26 molecular toxicity endpoints from invitroDB_ICE contained more than 500, 1000, and 2000 samples (chemicals), respectively, available for training. Although the invitroDB_ICE dataset was significantly smaller than the source data, it was, in turn, highly annotated. E.g., most chemical/endpoint pairs were annotated with a mechanistic target, which was derived manually from the combination of 'intended_target_family' and 'biological_process_target' in invitroDB. Currently, 67 mechanistic targets are assigned to the data, and the list is expanded continuously. Annotation of mechanistic targets is extremely useful for data applications as it facilitates linking endpoints to modes of action and, subsequently, to toxicological outcomes. Unless otherwise specified, invitroDB ICE data was used supplementary to the curated locally invitroDB.

**Molecular fingerprints.** In its simplest form, a structure-based molecular fingerprint is a binary vector of fixed length, a bit string of 0's and 1's that encodes the absence or presence of pre-defined substructures in a molecule.[15] An example, in PubChem fingerprints [881 bits], bit 121 encodes the presence of an unsaturated non-aromatic heteroatom-containing ring size 3. The most commonly used substructure-based fingerprints are Molecular ACCess Systems keys fingerprint (MACCS, 166-bit structural key set,[16]) and PubChem Fingerprints (PubChemFP, 881-bit structural key set,[17]). Other fingerprint ex-

amples include electrotopological state (E-state) fingerprints covering 79 E-state substructures as defined by Kier and Hall,[18] Klekota-Roth fingerprints (4860 bits/sub-structures),[19] and FP3/FP4 from Open Babel[20] (55 and 307 SMARTS patterns, respectively). Less intuitive than the substructure fingerprints are the topological path fingerprints, based on analyzing a molecule's fragments following a usually linear path up to a certain number of bonds (0-6) and then hashing every one of these paths to create one fingerprint with $n \cdot 1024$ bits. Examples of topological/path-based fingerprints include Daylight, Torsions, and Morgans fingerprints.[21] Molecular fingerprints are essential cheminformatics tools for virtual screening and mapping chemical space,[22] and the most commonly used fingerprinting algorithms can be found in various software packages. In this work, molecular fingerprints of compounds covered by invitroDB were used to train ML models to predict toxicity from the structure. After the curation, structures for the training of MLinvitroTox were converted into a set of structural and topological molecular fingerprints via CDK,[23] research development kit (RDkit),[24] OpenBabel,[20] Chemopy,[25] Padel,[26] and Pybel.[27,28] Although MACCs and PubChem fingerprints are most commonly used in computational toxicity,[29] there is little scientific evidence that these fingerprints yield optimal machine-learning outcomes. For the development of MLinvitroTox, the 23 types of fingerprints listed in Table ST1 were tested and evaluated for predicting activity endpoints covered in invitroDB.

In addition to the fingerprints from CDK and RDkit, ToxPrint (via ChemoTyper) and SIRIUS fingerprints (via Padel,[26] and Pybel[27,28]) were also generated and used for training and evaluation. ToxPrint fingerprints is a publicly-available set of structural keys targeting chemical chemotypes relevant for toxicity according to databases and regulatory inventories.[12,30,30–34] SIRIUS fingerprints is a compilation of structural fingerprints (MACCS, PubChem, OpenBabel, extended connectivity [ECFP], Klekota Roth, custom-made SMARTS, and ring systems, 3878 bits in positive and 4072 in negative mode, re-

Table ST1: Types of molecular fingerprints used for MLinvitroTox development. In total, 23 unique types were used. ECFP and FCFP types included all available radii, i.e., 0, 2, 4, and 6. S-structural fingerprints. T-topological fingerprints.

| Fingerprint | Package | Bits | Type | Fingerprint | Package | Bits | Type |
|---|---|---|---|---|---|---|---|
| Standard | CDK | 1024 | T | Extended | CDK | 1024 | Type |
| ECFP | CDK | 1024 | T | FCFP | CDK | 1024 | T |
| Estate | CDK | 79 | S | MACCS | CDK | 166 | S |
| PubChem | CDK | 881 | S | Morgan | RDkit | 1024 | T |
| Avalon | RDkit | 1024 | T | FeatMorgan | RDkit | 1024 | T |
| AtomPair | RDkit | 1024 | T | Torsion | RDkit | 1024 | T |
| RDkit | RDkit | 1024 | T | Layered | RDkit | 1024 | T |
| Pattern | RDkit | 1024 | T | ToxPrint | ChemoTyper | >700 | S |
| SIRIUS | Composite | 3878 (pos) | S | SIRIUS | Composite | 4072 (neg) | S |

spectively) used in the current work for the generation of molecular fingerprints from MS2 spectra (Figure SF1). CSI:FingerID is a state-of-the-art ML-based method for molecular structure annotation developed by the group of Sebastian Böcker from JENA and deployed as a web service integrated into the workflow of the software package SIRIUS (`https://bio.informatik.uni-jena.de/software/sirius/`).

# S3. MLinvitroTox Development

Figure SF4 shows ML workflows used in the current study. Model development was performed and optimized in two stages, varying data input (toxicity data curated according to 3 approaches and 23 types of molecular fingerprints) with ML parameters (29 models, 5 oversampling strategies, and 3 resampling strategies). The 500k modeling outcomes were used to define an optimal ML configuration for validation and testing with HRMS/MS data. At each stage, the models were optimized and retrained using hyperparameter tuning via random grid search (n=10-50) with nested resampling (folds=10) and tested on an independent test set (0.8/0.2 train/test). F-measure was used as the model performance metric. MS2 spectra and HRMS/MS raw data were converted into an appropriate format for validation and testing, processed to obtain a suitable mgf or msp format, converted into molecular fingerprints via SIRIUS, and used as input to MLinvitroTox models.

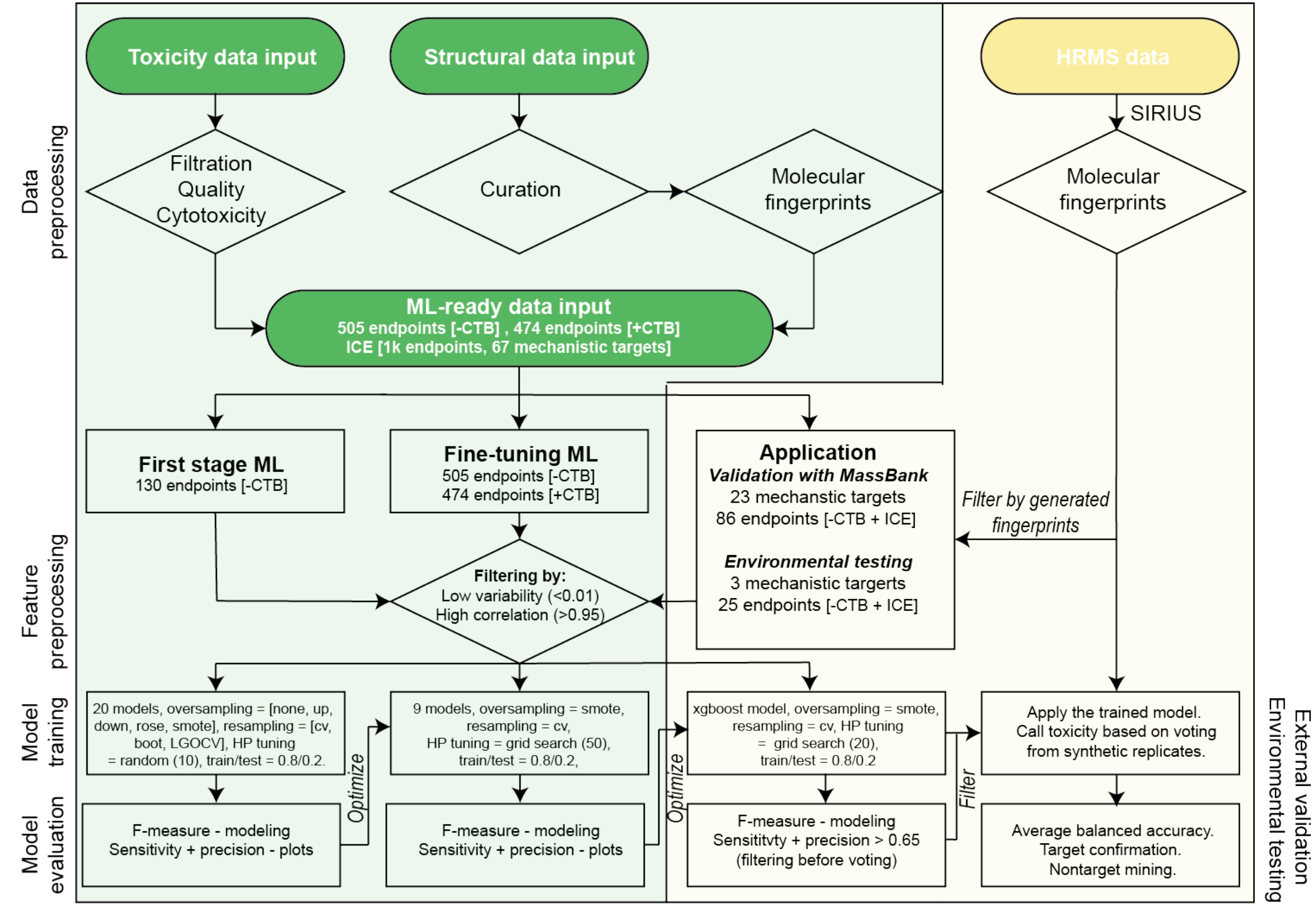


Figure SF4: ML workflows for model development (green) and validation/testing (yellow) used in the current study. Each workflow consisted of four main steps: (1) data preprocessing (2) feature preprocessing, (3) model training, and (4) model evaluation.

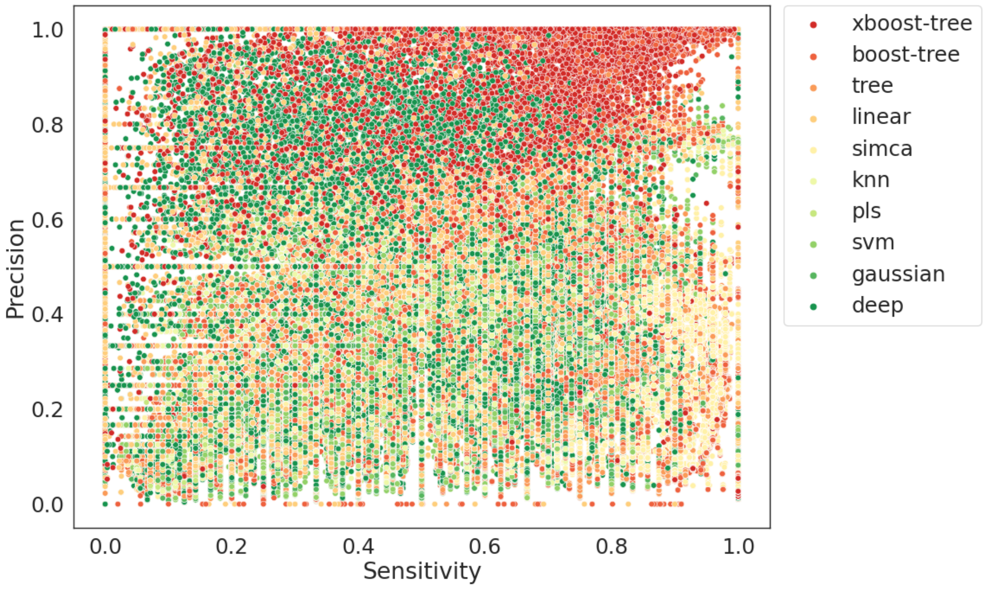


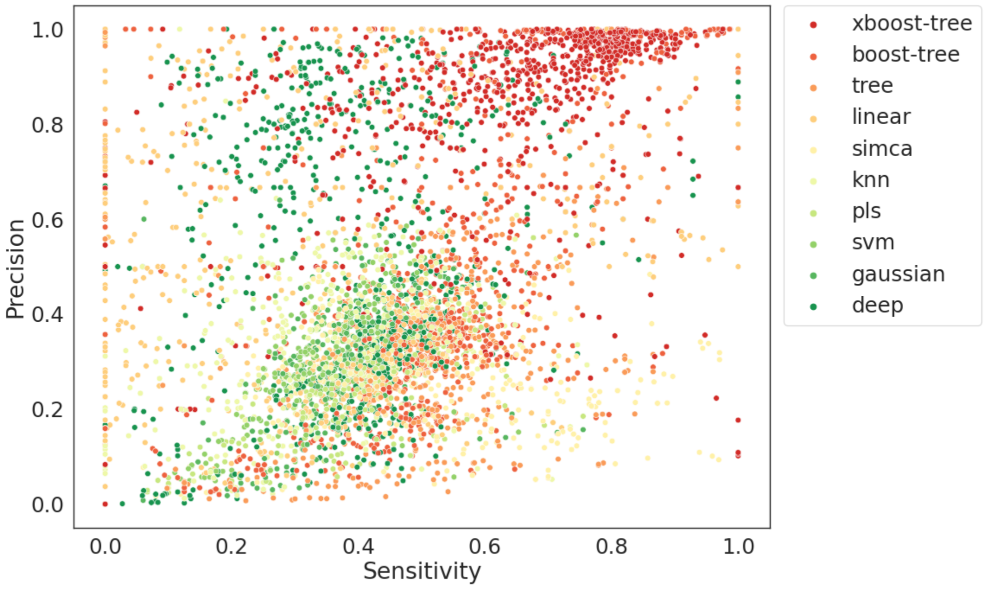


Figure SF5: Precision vs. sensitivity scatter plots for all (>500k) modeled combinations of endpoint/parameters (top) and mechanistic target/parameters (bottom, computed as an average across all relevant endpoints). The varied parameters included fingerprint, model, oversampling, resampling, and cytotoxicity. Both -CTB and +CTB data are represented. Combined initial modeling and fine-tuning results underline that xboost models yielded the most successful modeling outcomes for most endpoints as mechanistic targets.

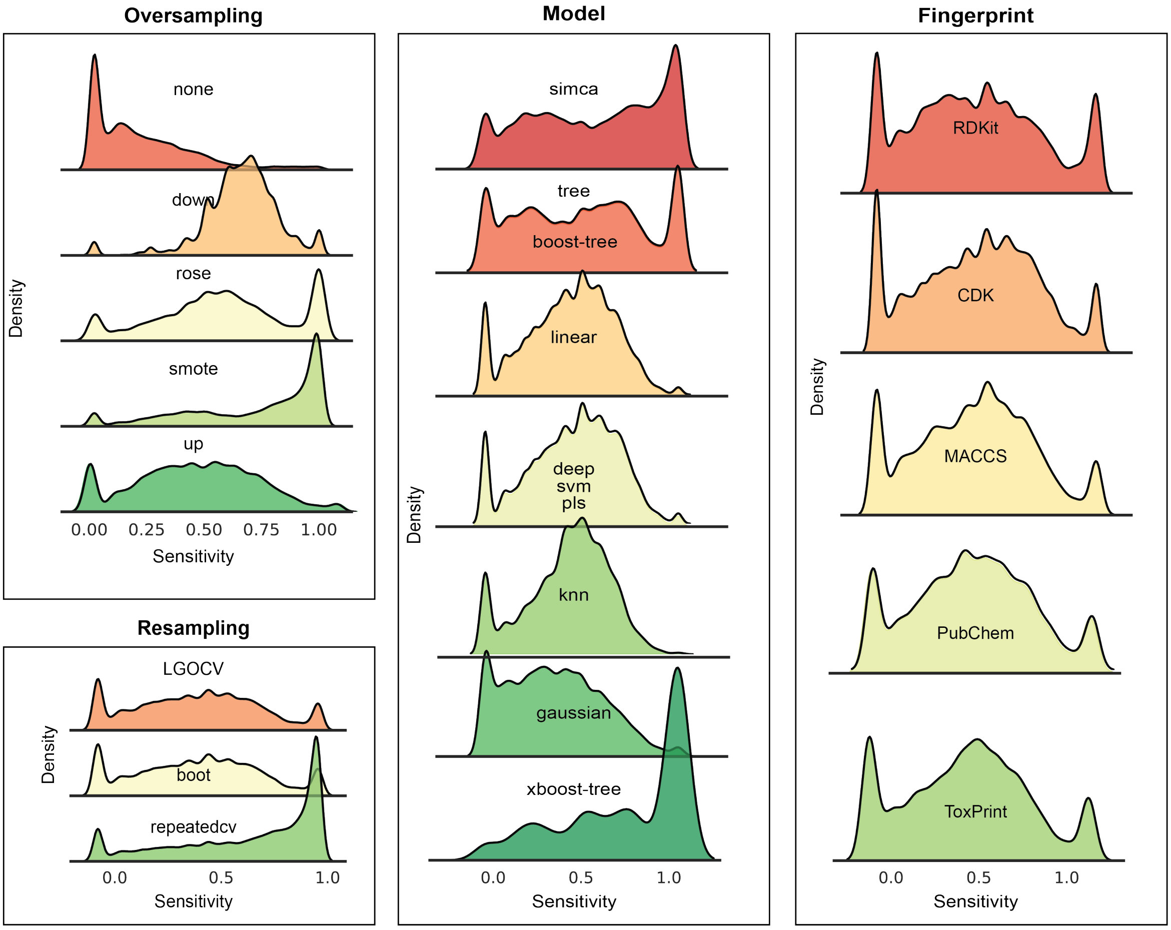


Figure SF6: Kernel density estimation provides a visual representation of sensitivity values distributions from the initial modeling phase when 20 model types were tested on 130 endpoints from the -CTB dataset, combined with varying 5 oversampling and 3 resampling techniques. Each subplot grouped the data by model, fingerprint, oversampling, and resampling. A high peak on a kernel density distribution plot indicates that the data is concentrated around a particular value. For example, a large peak for the "none" oversampling at a sensitivity value close to 0 on the x-axis would mean that all else being equal, this oversampling led to poor modeling results. A large peak for the "xboost-tree" model type (sensitivity close to 1) indicated a successful modeling configuration. Similarly, "repeatedcv" resampling generated on average models with the highest sensitivity values. There was no discernible difference in performance between the applied molecular fingerprints in the initial stages of modeling.

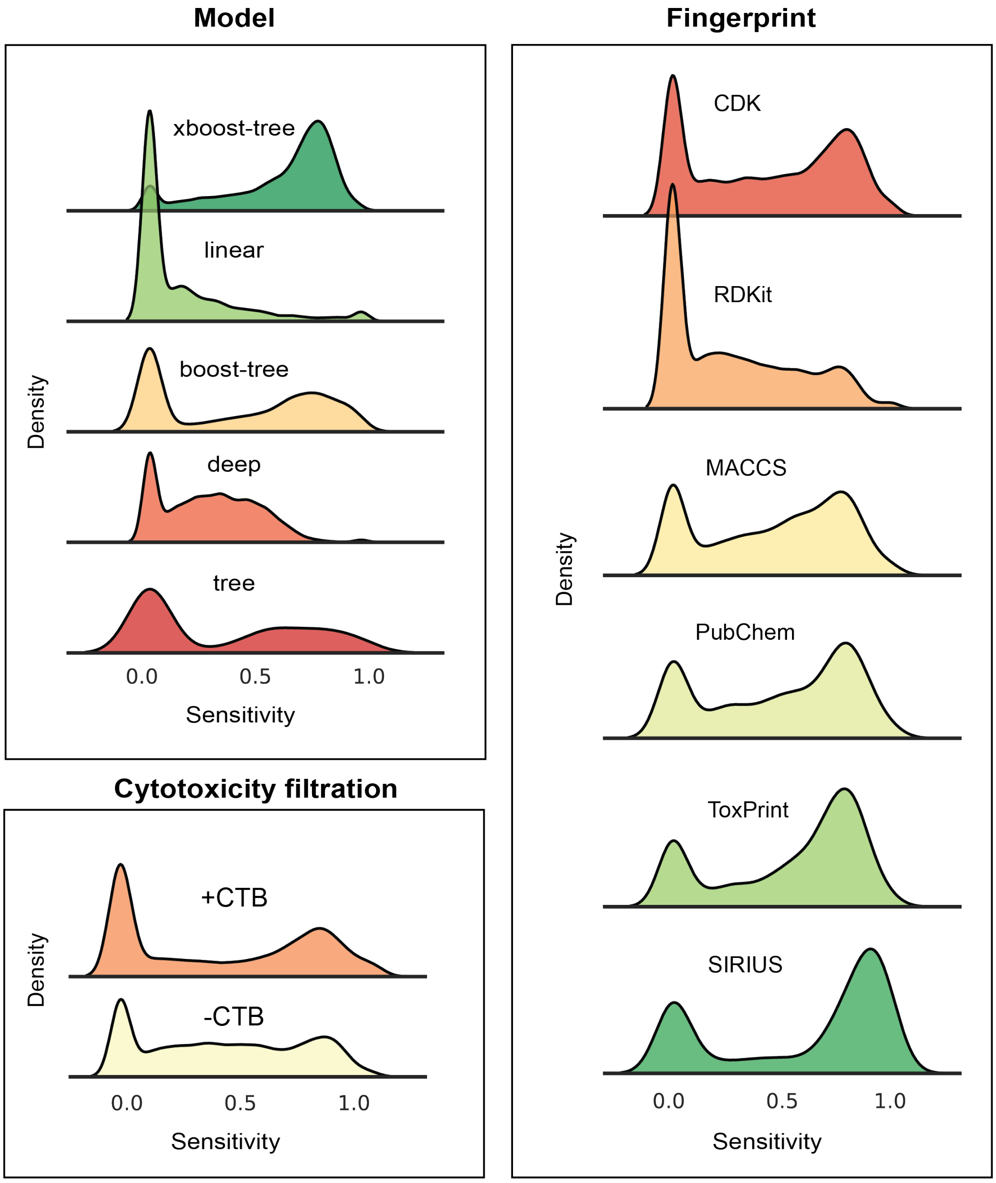


Figure SF7: Kernel density estimation provides a visual representation of sensitivity values distributions from the fine-tuning modeling phase when 9 models were optimized for 474 [+CTB dataset] and 505 [-CTB dataset] endpoints. The data were grouped by model, fingerprint, and cytotoxicity in each subplot. SMOTE oversampling and repeatedcv resampling were fixed during the initial stages of modeling. The results confirmed that xboost models and SIRIUS fingerprints yielded the best modeling outcomes. The effect of cytotoxicity filtering was not clear based on the current summary.

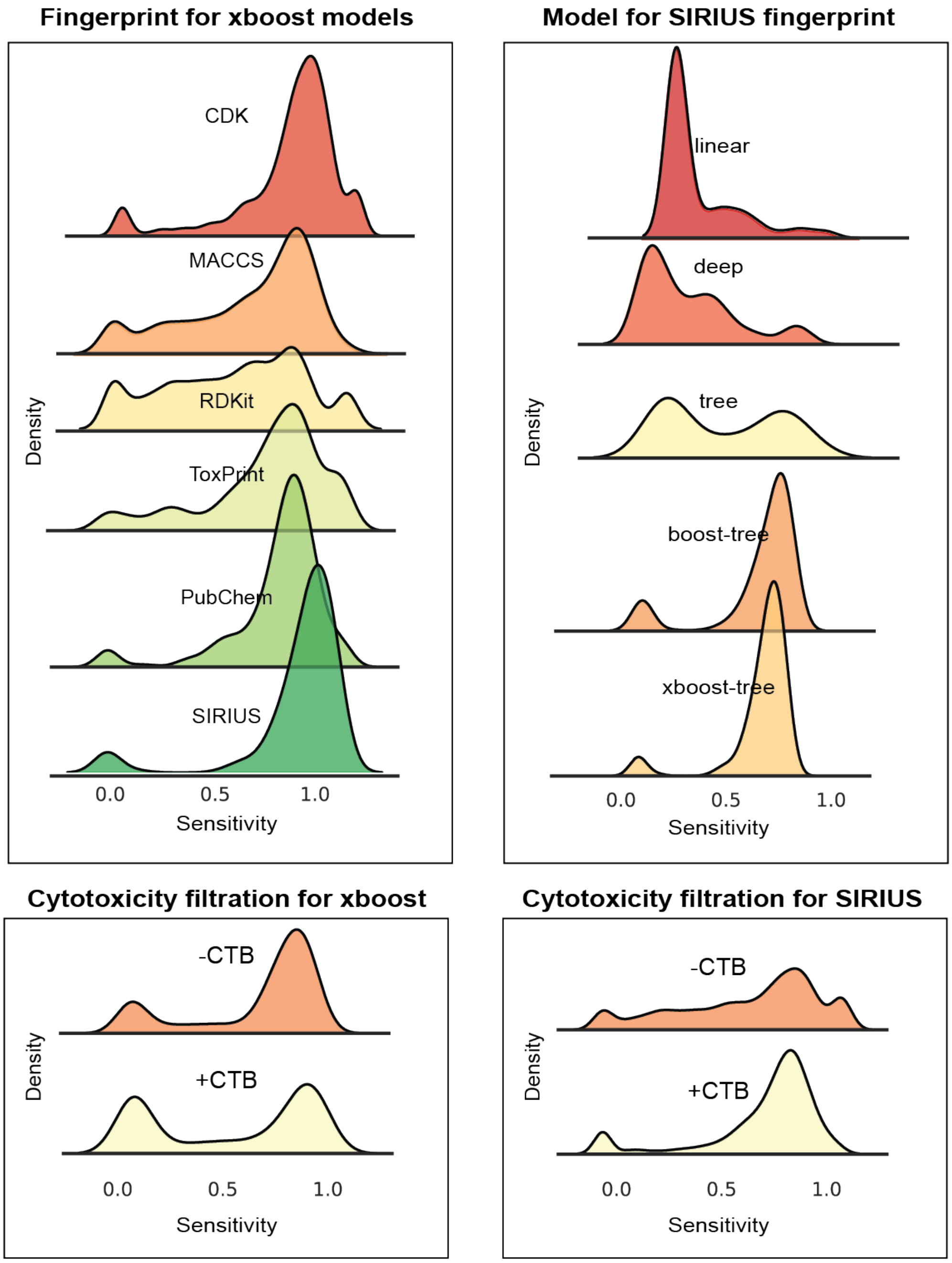


Figure SF8: Kernel density estimation provides a visual representation of sensitivity values distributions for models utilizing the optimal configuration [xboost, SIRIUS, SMOTE, repeatedcv] from both phases of modeling. As can be seen, with xboost, numerous molecular fingerprints performed well in modeling (high sensitivity values), and SIRIUS fingerprints yielded the best results with boosted-tree models. While for xboost models, cytotoxicity filtration seemed to influence the outcomes negatively; the opposite was true for SIRIUS fingerprints.

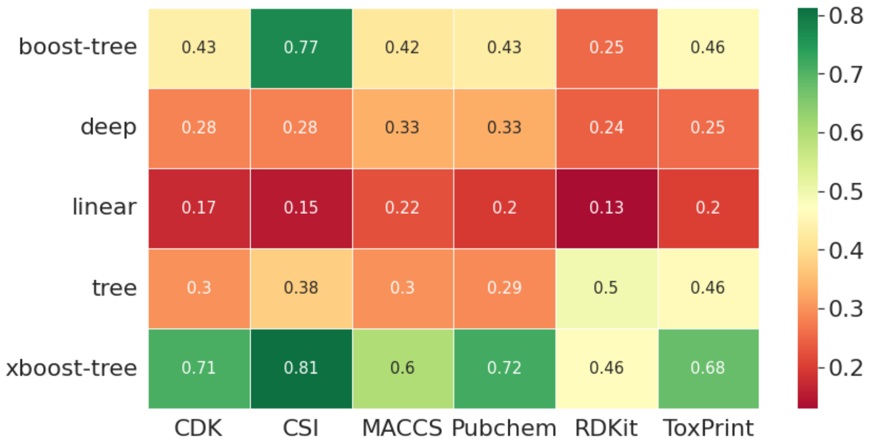


Figure SF9: Heat map with average sensitivity for fingerprint/model combinations with SMOTE oversampling and repeatedcv resampling. Both -CTB and +CTB data are represented. Combined results from the initial modeling and fine-tuning. The results show that the combination of SIRIUS fingerprints (here termed CSI) and xboost models yielded the best modeling outcomes.

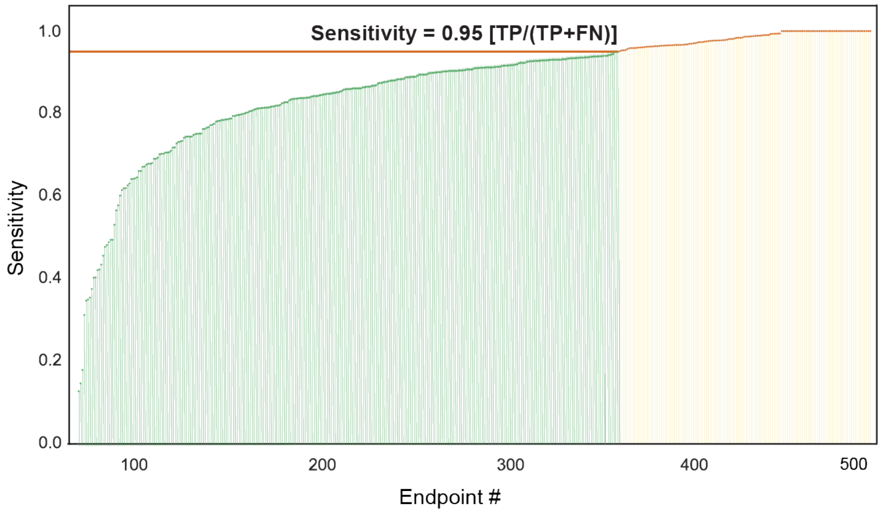


Figure SF10: Sensitivity distribution for the best model per endpoint across the studied parameters (fingerprint, model, oversampling, resampling, and cytotoxicity). Both -CTB and +CTB data are represented. Combined initial modeling and fine-tuning results show that 28% of the endpoints covering 24 mechanistic targets could be predicted with sensitivity > 0.95, providing the optimal input and modeling parameters. From the modeling point of view, a much larger number of endpoints/targets were predicted with a lower, yet acceptable, sensitivity, e.g., 0.75. Although an optimized set of parameters could be used for each endpoint, choosing the most successful set and using it across all endpoints is much more feasible.

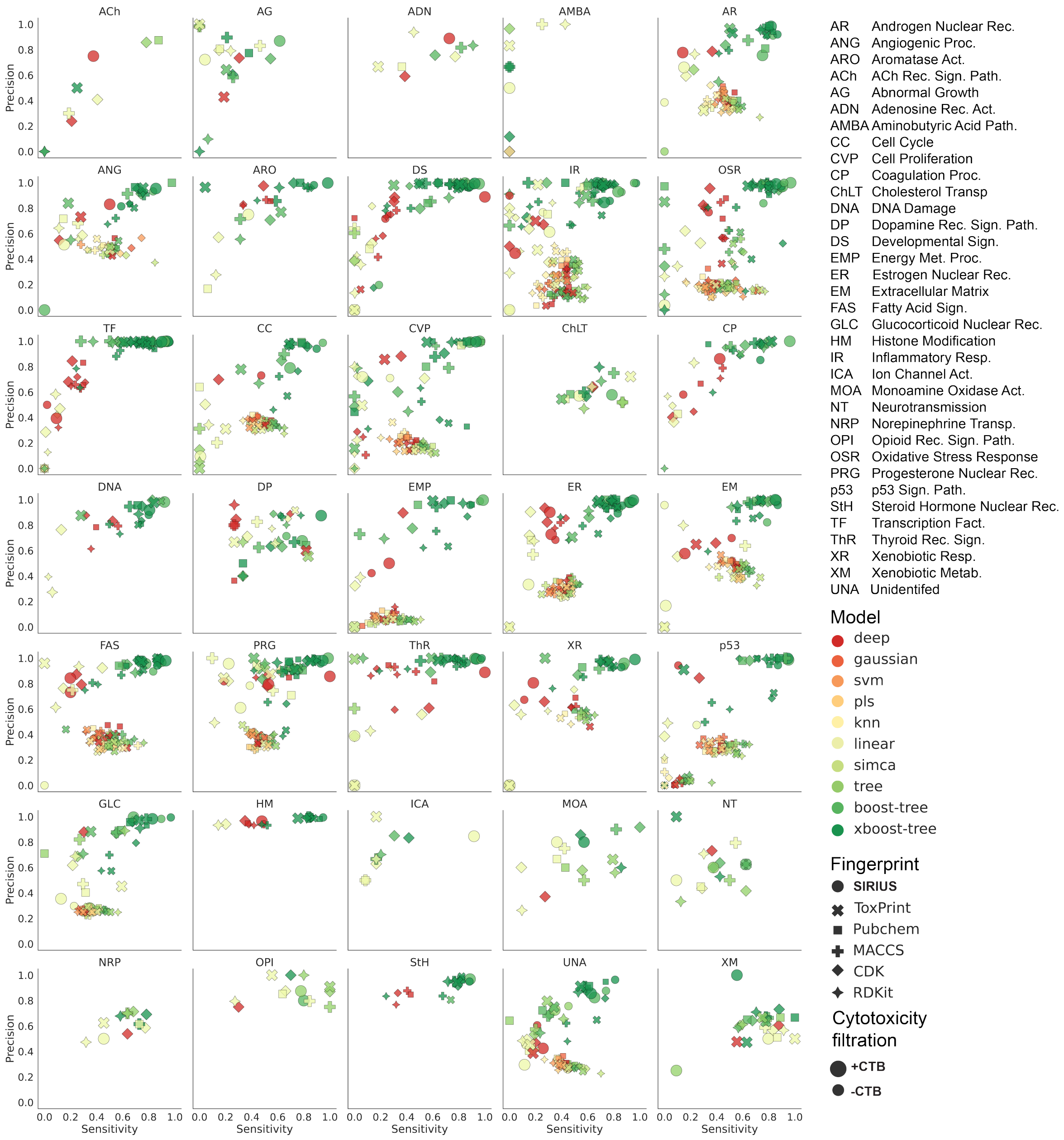


Figure SF11: Precision vs. sensitivity values from the MLinvitroTox modeling. In each plot, results for one mechanistic target are shown. Each data point represents the average metric across all relevant endpoints for a specific combination of parameters (input data [cytotoxicity], molecular fingerprint, model, oversampling, and resampling). Both -CTB and +CTB data are represented. Combined results from the initial modeling and fine-tuning.

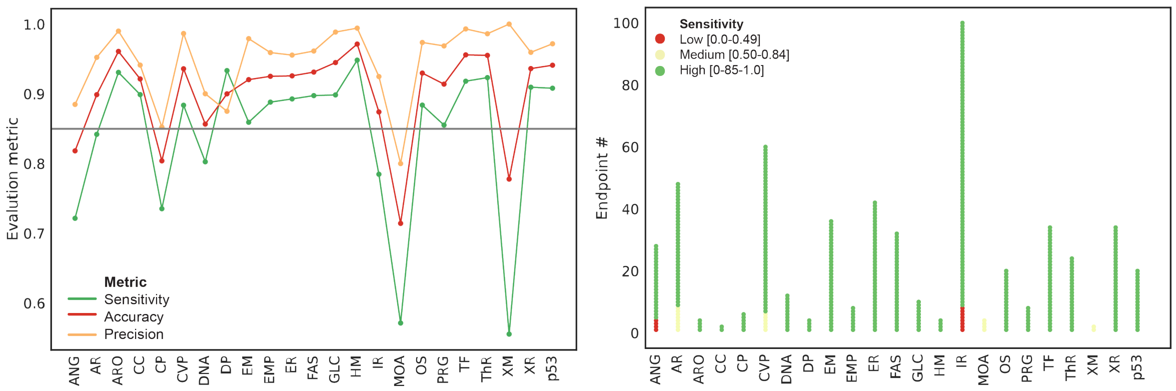


Figure SF12: Compilation of modeling outcomes for the optimal ML configuration [xboost-tree, SIRIUS fingerprint, smote oversampling, and repeatedcv resampling] (left) and Number of endpoints per mechanistic target, including sensitivity (right). Both -CTB and +CTB data are represented. Combined results from the initial modeling and fine-tuning. **ANG** Angiogenic process **AR** Androgen receptor **ARO** Aromatase activity **CC** Cell Cycle **CDP** Cell death process **CVP** Cell viability process **DNA** DNA damage **DS** Developmental signaling **EM** Extracellular matrix **EMP** Energy metabolism process **ER** Estrogen Receptor **FAS** Fatty Acid Signaling **GLC** Glucocorticoid Metabolic Process **HM** Histone Modification **IR** Inflammatory Response **MOA** Monoamine Oxidase Activity **NT** Neurotransmission **OSR** Oxidative Stress Response **PRG** Progesterone Receptor **TF** Transcription Factors **ThR** Thyroid Receptor **XM** Xenobiotic Metabolism **XR** Xenobiotic Responses **p53** p53 Pathway.

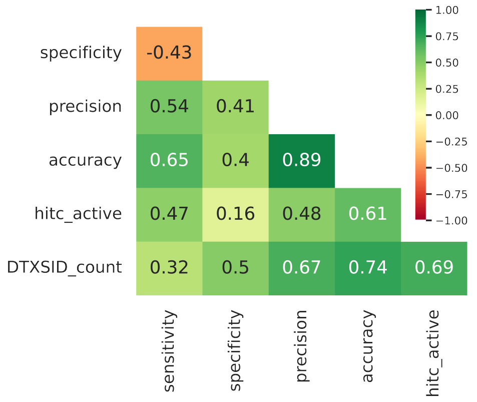


Figure SF13: Heat map showing correlation values between the obtained model metrics (sensitivity, specificity, precision, and accuracy with the number of chemicals and positive hit rates per endpoint). Both -CTB and +CTB data are represented. Combined results from the initial modeling and fine-tuning. As can be seen, the increasing number of samples available for training increased model performance, but the effect of toxic hit rates was much less prominent, indicating that the applied oversampling worked.

# S4. MassBank Validation

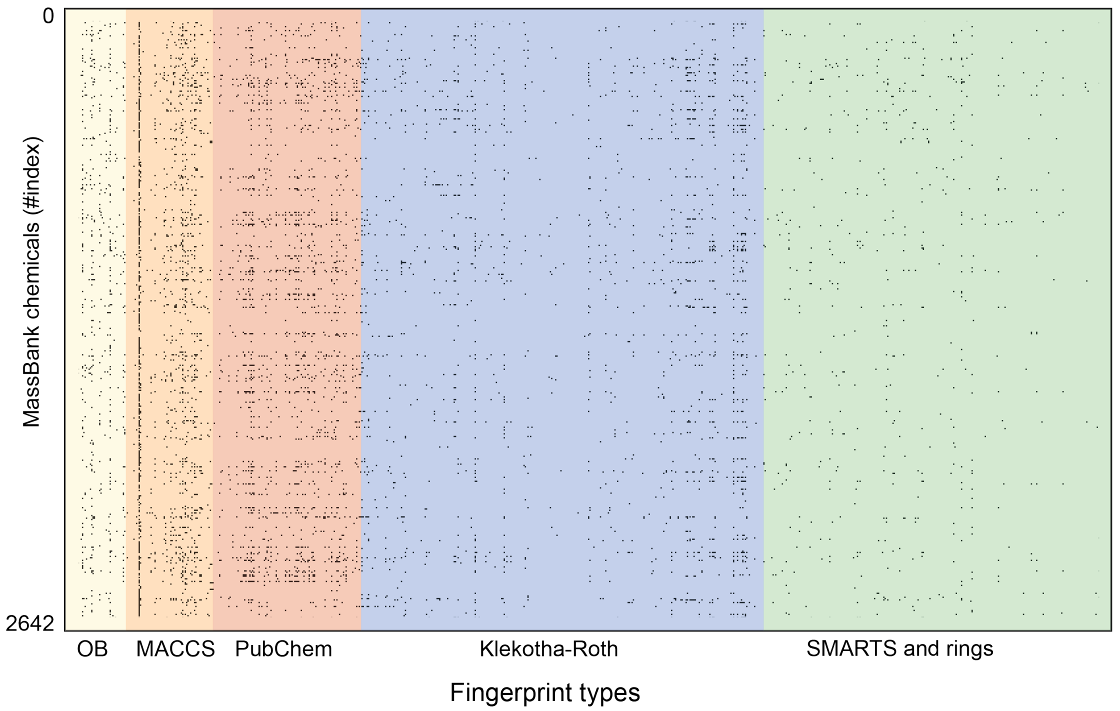


Figure SF14: A visual representation of the accuracy of the fingerprint prediction using SIRIUS for 2.5k compounds in MassBank. The black pixels represent incorrect predictions, i.e., disagreement between true [from structure via cheminformatic packages] and predicted [from SIRIUS] fingerprint bits. Although a certain divergence between the predicted and true fingerprints was observed (Figure SF14), the overall average accuracy for the prediction of the presence and absence of different substructures was satisfactory (balanced accuracy 98.5%, sensitivity 90.8%, Tanimoto coefficient 0.89) varying depending on the combination of quality of the input MS2 spectra and the intrinsic accuracy of SIRIUS predictors. The imperfect input for validation mimicked a real-life application of MLinvtroTox, where neither the structures nor the molecular fingerprints for unidentified HRMS/MS features are available. Despite the flawed input, MLinvitroTox exhibited a robust, balanced accuracy in predicting toxicity from both structures (0.75) and MS2 (0.74) for the MassBank compounds.

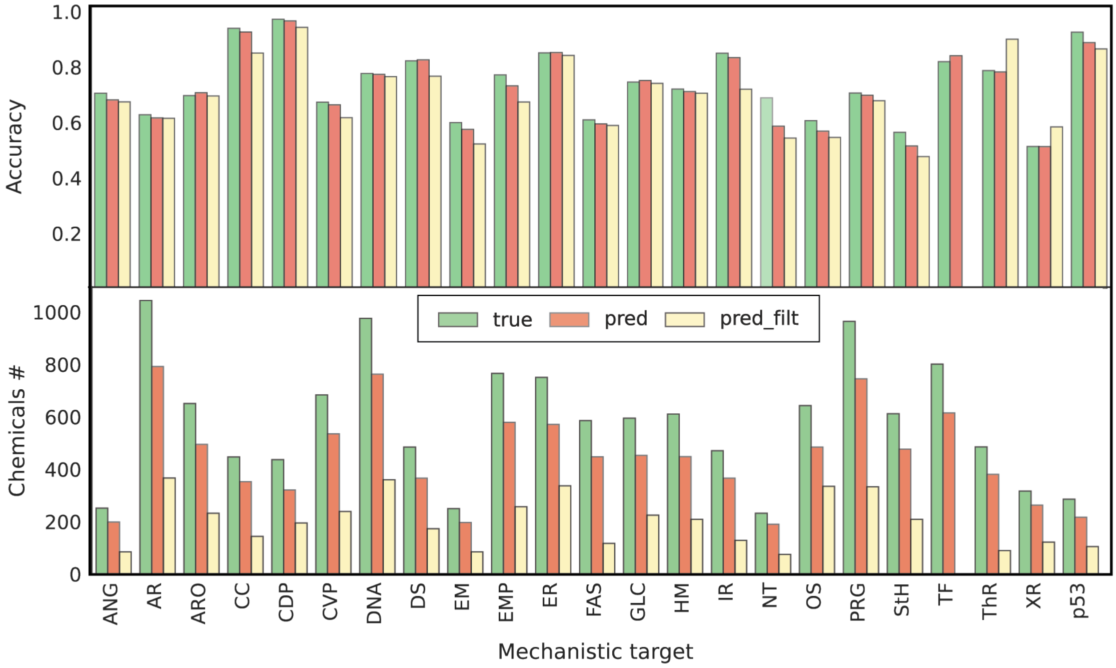


Figure SF15: Comparison of MLinvitroTox performance (balanced accuracy) for predicting toxic activities across 23 mechanistic targets for MassBank compounds from structures (True) and MS2 (Pred and Pred_filt). Pred_filt represents the subset of MassBank after removing compounds used as the SIRIUS training set. The number of chemicals available for validation in each group is in the bottom sub-figure. MLinvitroTox was trained on -CTB and ICE data with optimal ML parameters [xboost-tree, SIRIUS fingerprint, smote oversampling, and repeatedcv resampling] for each endpoint. The hit call for each chemical per endpoint was generated by voting of synthetic replicates. The shown results represent averages across endpoints for each mechanistic target. **ANG** Angiogenic process **AR** Androgen receptor **ARO** Aromatase **CC** Cell cycle **CDP** Cell death **CVP** Cell viability **DNA** DNA damage **DS** Developmental signaling **EM** Extracellular matrix **EMP** Energy metabolism **ER** Estrogen receptor **FAS** Fatty acid signaling **GLC** Glucocorticoid receptor **HM** Histone modification **IR** Inflammatory response **MOA** Monoamine oxidase **NT** Neurotransmission **OSR** Oxidative stress response **PRG** Progesterone receptor **StH** Steroid receptor **TF** Transcription factors **ThR** Thyroid receptor **XM** Xenobiotic response **p53** p53 pathway.

# S5. Environmental Application

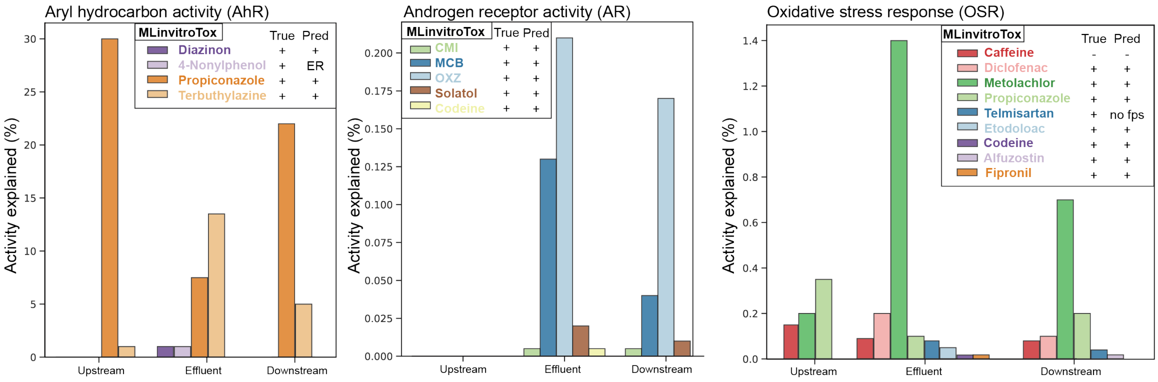


Figure SF16: A visual representation of the accuracy of MLinvitroTox predictions for targets linked by Neale et al.[35] to toxic effects in AhR, AR, or OSR endpoints. Nomenclature: + correct prediction, - wrong prediction, no fps - no fps generated, ER - estrogenic compounds not covered in the current analysis. MCB - moclobemide, OXZ - oxazepam, CMI - 5-Chloro-2-methyl-4-isothiazolin-3-on. For AR and AhR the relevant target compounds were all predicted correctly from true and predicted fingerprints. For OSR, the toxicity of 13 out of 24 compounds, including 7 out of 9 of the most potent ones, was predicted correctly. The toxicity could not be confirmed for caffeine via either the true or the predicted fingerprints. For telmisartan, no molecular fingerprints were generated. Out of the 24 OSR-active compounds from the original analysis, molecular fingerprints were predicted by SIRIUS for 18 (13 in positive and 5 in negative mode).

Table ST2: List of targets from the environmental application predicted correctly based on the obtained HRMS/MS spectra. CMI - 5-Chloro-2-methyl-4-isothiazolin-3-on.

| Effect | Compound | Effect | Compound | Effect | Compound |
|---|---|---|---|---|---|
| AhR | Terbutylazin | AR | Codein | OSR | Metolachlor |
| AhR | Diazinon | AR | Moclobemid | OSR | Cyprodinil |
| AhR | Propiconazol | AR | Sotalol | OSR | Mefenamic acid |
| AR | CMI | AR | Oxazepam | OSR | Diclofenac |
| OSR | Alfuzosin | OSR | Fipronil | OSR | Propiconazol |
| OSR | Codein | OSR | Etodolac | OSR | Difenoconazol |
| OSR | Penconazol | OSR | Azoxystrobin | OSR | Terbutryn |

Table ST3: Identities of the probable structures obtained from automatic MS-DIAL matches (threshold 0.85) with MassBank spectral records. The last column (DB) indicates whether the corresponding invitroDB records were available.

| stdinchikey | dtxsid | casrn | name | effect | DB |
|---|---|---|---|---|---|
| AFDXODALSZRGIH-UHFFFAOYSA-N | DTXSID4048147 | 830-09-1 | 4-Methoxycinnamic acid | AR | no |
| ASOKPJOREAFHNY-UHFFFAOYSA-N | DTXSID3044627 | 2592-95-2 | 1-Hydroxybenzotriazole | AhR, AR | yes |
| ATEFPOUAMCWAQS-UHFFFAOYSA-N | DTXSID00197560 | 486-35-1 | Daphnetin | AR | no |
| BACYUWVYYTXETD-UHFFFAOYSA-N | DTXSID7042011 | 97-78-9 | N-Dodecanoyl-N-methylglycine | AhR, AR | yes |
| BAYGVMXZJBFEMB-UHFFFAOYSA-N | DTXSID2075392 | 402-45-9 | 4-(Trifluoromethyl)phenol | AhR, AR | no |
| BMPDWHIDQYTSHX-UHFFFAOYSA-N | DTXSID50865484 | 29331-92-8 | 10-hydroxycarbazepine | AhR, AR | no |
| BNCADMBVWNPPIZ-UHFFFAOYSA-N | DTXSID9027520 | 3089-11-0 | Hexa(methoxymethyl)melamine | AhR, AR, OSR | no |
| BTJIUGUIPKRLHP-UHFFFAOYSA-N | DTXSID0021834 | 100-02-7 | 4-Nitrophenol | AhR, AR, OSR | yes |
| BWHOZHOGCMHOBV-BQYQJAHWSA-N | DTXSID1031626 | 1896-62-4 | Methyl trans-styryl ketone | AR | no |
| BWLBGMIXKSTLSX-UHFFFAOYSA-N | DTXSID4032954 | 594-61-6 | 2-Hydroxyisobutyric acid | AR, OSR | no |
| CDAWCLOXVUBKRW-UHFFFAOYSA-N | DTXSID8024498 | 95-55-6 | 2-Aminophenol | AhR, AR | yes |
| CMGDVUCDZOBDNL-UHFFFAOYSA-N | DTXSID50274037 | 29878-31-7 | 4-Methyl-1,2,3-benzotriazole | AhR, AR | no |
| COLNVLDHVKWLRT-QMMMGPOBSA-N | DTXSID4040763 | 63-91-2 | L-Phenylalanine | AhR, AR | no |
| CPHJEACXPATRSU-UHFFFAOYSA-N | DTXSID5044865 | 18924-66-8 | 2,2′-(Tetradecylimino)diethanol | AR | yes |
| CTTHWASMBLQOFR-UHFFFAOYSA-N | DTXSID7057945 | 120923-37-7 | Amidosulfuron | AhR, AR | no |
| CXVGEDCSTKKODG-UHFFFAOYSA-N | DTXSID2042436 | 4065-45-6 | Sulisobenzone | AhR, AR, OSR | yes |
| CYESCLHCWJKRKM-UHFFFAOYSA-N | DTXSID2041468 | 2327-02-8 | 3,4-Dichlorophenylurea | AhR, AR | yes |
| DHHVAGZRUROJKS-UHFFFAOYSA-N | DTXSID9023461 | 122-09-8 | Phentermine | AR | no |
| DLNKOYKMWOXYQA-IONNQARKSA-N | DTXSID50889347 | 492-39-7 | Benzenemethanol (long name) | AR | no |
| DMSMPAJRVJJAGA-UHFFFAOYSA-N | DTXSID5032523 | 2634-33-5 | 1,2-Benzisothiazolin-3-one | AhR, AR, OSR | yes |
| DOOTYTYQINUNNV-UHFFFAOYSA-N | DTXSID0040701 | 77-93-0 | Triethyl citrate | AR | no |
| DVBDYPDVNRJKNJ-UHFFFAOYSA-N | DTXSID30891443 | 120375-14-6 | Metolachlor morpholinone | AhR, AR, OSR | yes |
| DZBUGLKDJFMEHC-UHFFFAOYSA-N | DTXSID8059766 | 260-94-6 | Acridine | AhR, AR | no |
| FDGQSTZJBFJUBT-UHFFFAOYSA-N | DTXSID8045983 | 68-94-0 | Hypoxanthine | AR | no |
| FIQMHBFVRAXMOP-UHFFFAOYSA-N | DTXSID2022121 | 791-28-6 | Triphenylphosphine oxide | AhR, AR | no |
| FMJSMJQBSVNSBF-UHFFFAOYSA-N | DTXSID9025299 | 6197-30-4 | 2-Ethylhexyl-2-cyano-3,3-diphenylacrylate | AhR, AR | yes |
| FNCMBMZOZQAWJA-UHFFFAOYSA-N | DTXSID8042139 | 2635-10-1 | Methiocarb sulfoxide | AR | no |
| FQXWEKADCSXYOC-UHFFFAOYSA-N | DTXSID50869644 | 120067-83-6 | Fipronil sulfide | AhR, AR | no |
| GCKLGRUZDXSATG-UHFFFAOYSA-N | DTXSID80173802 | 19988-24-0 | Triazinone (long name) | AR | no |

**Table ST3 continued from previous page**

| stdinchikey | dtxsid | casrn | preferredName | effect | DB |
|---|---|---|---|---|---|
| GSDSWSVVBLHKDQ-JTQLQIEISA-N | DTXSID0041060 | 100986-85-4 | Levofloxacin | AhR, AR, OSR | no |
| HCFDWZZGGLSKEP-UHFFFAOYSA-N | DTXSID1022970 | 469-21-6 | Doxylamine | AR | no |
| HLFSDGLLUJUHTE-SNVBAGLBSA-N | DTXSID4023206 | 14769-73-4 | Levamisole | AR | no |
| HRKAMJBPFPHCSD-UHFFFAOYSA-N | DTXSID8040698 | 126-71-6 | Triisobutyl phosphate | AR | no |
| IAOZJIPTCAWIRG-UHFFFAOYSA-N | DTXSID90274359 | 25548-16-7 | Methyl alpha-aspartylphenylalaninate | AhR, AR | no |
| IBGBGRVKPALMCQ-UHFFFAOYSA-N | DTXSID4074512 | 139-85-5 | Benzaldehyde, 3,4-dihydroxy- | AhR, AR | no |
| IIPZYDQGBIWLBU-UHFFFAOYSA-N | DTXSID7041883 | 1420-07-1 | Dinoterb | AR, OSR | yes |
| ILEDWLMCKZNDJK-UHFFFAOYSA-N | DTXSID3075383 | 305-01-1 | Esculetin | AhR, AR, OSR | no |
| IOJUPLGTWVMSFF-UHFFFAOYSA-N | DTXSID7024586 | 95-16-9 | Benzothiazole | AhR, AR | yes |
| ISZQNKFXNXQTTF-NACSPRHISA-N | DTXSID50892513 | 252913-85-2 | Trifloxystrobin acid | AhR, AR, OSR | no |
| IVDOUUOLLFEMJQ-UHFFFAOYSA-N | DTXSID90891484 | 1076199-80-8 | Cetirizine N-oxide | AhR, AR | no |
| IYWFOPPYQUEKHN-UHFFFAOYSA-N | DTXSID70707852 | 493011-70-4 | 5-(2-Phenylethenyl)benzene-1,2,3-triol | AR | no |
| JAWPQJDOQPSNIQ-UHFFFAOYSA-N | DTXSID40215070 | 64744-50-9 | Gabapentin-lactam | AR | no |
| JLFNLZLINWHATN-UHFFFAOYSA-N | DTXSID4027579 | 4792-15-8 | Pentaethylene glycol | AR | no |
| JLKIGFTWXXRPMT-UHFFFAOYSA-N | DTXSID8026064 | 723-46-6 | Sulfamethoxazole | AR | no |
| JOFDPSBOUCXJCC-UHFFFAOYSA-N | DTXSID6037697 | 60397-77-5 | N-(2,4-Dimethylphenyl)formamide | AhR, AR | no |
| JPMIIZHYYWMHDT-UHFFFAOYSA-N | DTXSID1025805 | 26530-20-1 | Octhilinone | AR | yes |
| JTZCTMAVMHRNTR-UHFFFAOYSA-N | DTXSID2032639 | 55512-33-9 | Pyridate | AhR, AR | no |
| KMAKOBLIOCQGJP-UHFFFAOYSA-N | DTXSID50227886 | 771-50-6 | Indole-3-carboxylic acid | AhR, AR | no |
| KVBGVZZKJNLNJU-UHFFFAOYSA-N | DTXSID5044788 | 120-18-3 | Naphthalene-2-sulfonic acid | AhR, AR, OSR | yes |
| KWGRBVOPPLSCSI-WPRPVWTQSA-N | DTXSID0022985 | 299-42-3 | Ephedrine | AR | no |
| LEZHOZPJYAQQNU-UVTDQMKNSA-N | DTXSID60583566 | 676228-91-4 | Thiacloprid amide | AhR, AR | no |
| LJKAKWDUZRJNPJ-UHFFFAOYSA-N | DTXSID50142848 | 100-90-3 | N(4)-Acetylsulfamethazine | AhR, AR | no |
| LLIKIPAUZJTRGB-UHFFFAOYSA-N | DTXSID60747025 | 71676-01-2 | Aminomethoxybenzamide (long name) | AhR, AR, OSR | no |
| LOUPRKONTZGTKE-LHHVKLHASA-N | DTXSID4023549 | 56-54-2 | Quinidine | AR, OSR | no |
| MESJRHHDBDCQTH-UHFFFAOYSA-N | DTXSID0025101 | 99-07-0 | 3-Dimethylaminophenol | AR | yes |
| MIWRSUQXSCLDNV-UHFFFAOYSA-N | DTXSID0052839 | 35045-02-4 | Metribuzin-DA | AR | no |
| MKXKFYHWDHIYRV-UHFFFAOYSA-N | DTXSID7032004 | 13311-84-7 | Flutamide | AhR, AR, OSR | yes |
| MPDGHEJMBKOTSU-YKLVYJNSSA-N | DTXSID9020669 | 471-53-4 | Glycyrrhetinic acid | AhR, AR | no |
| MTKNGOHFNXIVOS-UHFFFAOYSA-N | DTXSID80202141 | 53885-35-1 | Ticlopidine hydrochloride | AR | no |
| MZBFYGYTZIZFQC-UHFFFAOYSA-N | DTXSID00891339 | NOCAS_891339 | Tetradecane-7-sulfonic acid | AhR, AR, OSR | no |
| NDAUXUAQIAJITI-UHFFFAOYSA-N | DTXSID5021255 | 18559-94-9 | Albuterol | AR, OSR | no |

**Table ST3 continued from previous page**

| stdinchikey | dtxsid | casrn | preferredName | effect | DB |
|---|---|---|---|---|---|
| NFMIMWNQWAWNDW-UHFFFAOYSA-N | DTXSID6037807 | 2163-68-0 | 2-Hydroxyatrazine | AR | no |
| NGBFQHCMQULJNZ-UHFFFAOYSA-N | DTXSID2023690 | 56211-40-6 | Torsemide | AhR, AR, OSR | no |
| NJIZUWGMNCUKGU-UHFFFAOYSA-N | DTXSID00155362 | 127-74-2 | N(4)-Acetylsulfadiazine | AhR, AR, OSR | no |
| NLDDIKRKFXEWBK-AWEZNQCLSA-N | DTXSID3041035 | 23513-14-6 | (6)-Gingerol | AhR, AR | no |
| NQPDXQQQCQDHHW-UHFFFAOYSA-N | DTXSID7043952 | 68786-66-3 | Triclabendazole | AhR, AR | yes |
| NTDQQZYCCIDJRK-UHFFFAOYSA-N | DTXSID9022312 | 1806-26-4 | 4-Octylphenol | AhR, AR, OSR | yes |
| OISVCGZHLKNMSJ-UHFFFAOYSA-N | DTXSID7051557 | 108-48-5 | 2,6-Dimethylpyridine | AhR, AR | no |
| OLNJUISKUQQNIM-UHFFFAOYSA-N | DTXSID5060069 | 487-89-8 | 1H-Indole-3-carboxaldehyde | AhR, AR | no |
| OUSYWCQYMPDAEO-UHFFFAOYSA-N | DTXSID30190483 | 36993-94-9 | 2H-1,2,4-triazin-5-one, 3-methyl-6-phenyl- | AhR, AR | no |
| OWRCNXZUPFZXOS-UHFFFAOYSA-N | DTXSID3025178 | 102-06-7 | 1,3-Diphenylguanidine | AhR, AR | yes |
| PHNUZKMIPFFYSO-UHFFFAOYSA-N | DTXSID2020420 | 23950-58-5 | Propyzamide | AR | yes |
| PMZDQRJGMBOQBF-UHFFFAOYSA-N | DTXSID50209980 | 611-36-9 | 4-Hydroxyquinoline | AhR | no |
| PORQOHRXAJJKGK-UHFFFAOYSA-N | DTXSID5032315 | 64359-81-5 | 4,5-Dichloro-2-octyl-3(2H)-isothiazolone | AhR, AR | yes |
| PROQIPRRNZUXQM-ZXXIGWHRSA-N | DTXSID9022366 | 50-27-1 | Estriol | AR | yes |
| PVNIIMVLHYAWGP-UHFFFAOYSA-N | DTXSID1020932 | 59-67-6 | Nicotinic acid | AhR, AR | no |
| QHUWVQWAKAJLTJ-UHFFFAOYSA-N | DTXSID80895138 | 314020-44-5 | Oxadiazepinedione (long name) | AR | no |
| QNBTYORWCCMPQP-JXAWBTAJSA-N | DTXSID101017883 | 113210-98-3 | (Z)-Dimethomorph | AhR, AR | no |
| QRUDEWIWKLJBPS-UHFFFAOYSA-N | DTXSID6020147 | 95-14-7 | 1,2,3-Benzotriazole | AhR, AR | yes |
| RLFWWDJHLFCNIJ-UHFFFAOYSA-N | DTXSID8048860 | 83-07-8 | Ampyrone | AhR, AR | no |
| RTCOGUMHFFWOJV-UHFFFAOYSA-N | DTXSID6034764 | 111991-09-4 | Nicosulfuron | AhR, AR | no |
| RWZYAGGXGHYGMB-UHFFFAOYSA-N | DTXSID8020094 | 118-92-3 | Anthranilic acid | AR | no |
| SBNFWQZLDJGRLK-UHFFFAOYSA-N | DTXSID7032688 | 26002-80-2 | Phenothrin | AR | yes |
| SECXISVLQFMRJM-UHFFFAOYSA-N | DTXSID6020856 | 872-50-4 | N-Methyl-2-pyrrolidone | AR | no |
| SONNWYBIRXJNDC-VIFPVBQESA-N | DTXSID9023465 | 59-42-7 | Phenylephrine | AR | no |
| SOQJPQZCPBDOMF-YCUXZELOSA-N | DTXSID2022670 | 22298-29-9 | Betamethasone benzoate | AR, OSR | no |
| SYELZBGXAIXKHU-UHFFFAOYSA-N | DTXSID1020514 | 1643-20-5 | N,N-Dimethyldodecylamine-N-oxide | AR | no |
| TYBHZVUFOINFDV-UHFFFAOYSA-N | DTXSID90165596 | 15435-29-7 | Bromochlorophene | AhR, AR, OSR | no |
| TYEYBOSBBBHJIV-UHFFFAOYSA-N | DTXSID9060524 | 600-18-0 | 2-Oxobutyric acid | AR, OSR | no |
| TZBJGXHYKVUXJN-UHFFFAOYSA-N | DTXSID5022308 | 446-72-0 | Genistein | AhR, AR | yes |
| UFFBMTHBGFGIHF-UHFFFAOYSA-N | DTXSID8026307 | 87-62-7 | 2,6-Dimethylaniline | AhR, AR | yes |
| UHGULLIUJBCTEF-UHFFFAOYSA-N | DTXSID1024467 | 136-95-8 | 2-Aminobenzothiazole | AhR, AR, OSR | yes |
| VHYCDWMUTMEGQY-UHFFFAOYSA-N | DTXSID6022682 | 66722-44-9 | Bisoprolol | AR | no |

**Table ST3 continued from previous page**

| stdinchikey | dtxsid | casrn | preferredName | effect | DB |
|---|---|---|---|---|---|
| WIYOSEJTAADVPJ-UHFFFAOYSA-N | DTXSID10204077 | 555-25-9 | Acetamide (long name) | AhR, AR | no |
| WPYMKLBDIGXBTP-UHFFFAOYSA-N | DTXSID6020143 | 65-85-0 | Benzoic acid | AR | no |
| WUFQLZTXIWKION-UHFFFAOYSA-N | DTXSID40197807 | 495-59-0 | 3-Deoxyvasicine | AhR, AR | no |
| WXNZTHHGJRFXKQ-UHFFFAOYSA-N | DTXSID1021871 | 106-48-9 | 4-Chlorophenol | AR | no |
| XLMALTXPSGQGBX-GCJKJVERSA-N | DTXSID1023524 | 469-62-5 | Propoxyphene | AR | no |
| XPAZGLFMMUODDK-UHFFFAOYSA-N | DTXSID8020965 | 94-52-0 | 6-Nitrobenzimidazole | AhR, AR | no |
| XSAYZAUNJMRRIR-UHFFFAOYSA-N | DTXSID2041389 | 93-08-3 | 2′-Acetonaphthone | AR | yes |
| XUJNEKJLAYXESH-REOHCLBHSA-N | DTXSID8022876 | 52-90-4 | L-Cysteine | AhR, AR | no |
| YEDUAINPPJYDJZ-UHFFFAOYSA-N | DTXSID6061315 | 934-34-9 | Benzothiazolone | AhR, AR, OSR | no |
| YKFLAYDHMOASIY-UHFFFAOYSA-N | DTXSID6041210 | 99-85-4 | gamma-Terpinene | AhR, AR | no |
| YUVKUEAFAVKILW-UHFFFAOYSA-N | DTXSID3058163 | 69335-91-7 | Fluazifop | AhR, AR | no |
| YXIWHUQXZSMYRE-UHFFFAOYSA-N | DTXSID1020807 | 149-30-4 | 2-Mercaptobenzothiazole | AhR, AR, OSR | yes |
| ZCGLNFAUVVHTQO-UHFFFAOYSA-N | DTXSID80891493 | 440341-75-3 | 3-[(4-chlorobenzoyl)amino]propanoic acid | AhR, AR | no |
| ZFXYFBGIUFBOJW-UHFFFAOYSA-N | DTXSID5021336 | 58-55-9 | Theophylline | AR | yes |
| ZHBBDQFXEDCQFI-UHFFFAOYSA-N | DTXSID20865052 | 22454-92-8 | Mesurol phenol sulfoxide | AhR, AR | no |
| ZUHZGEOKBKGPSW-UHFFFAOYSA-N | DTXSID7044396 | 143-24-8 | 2,5,8,11,14-Pentaoxapentadecane | AR | no |

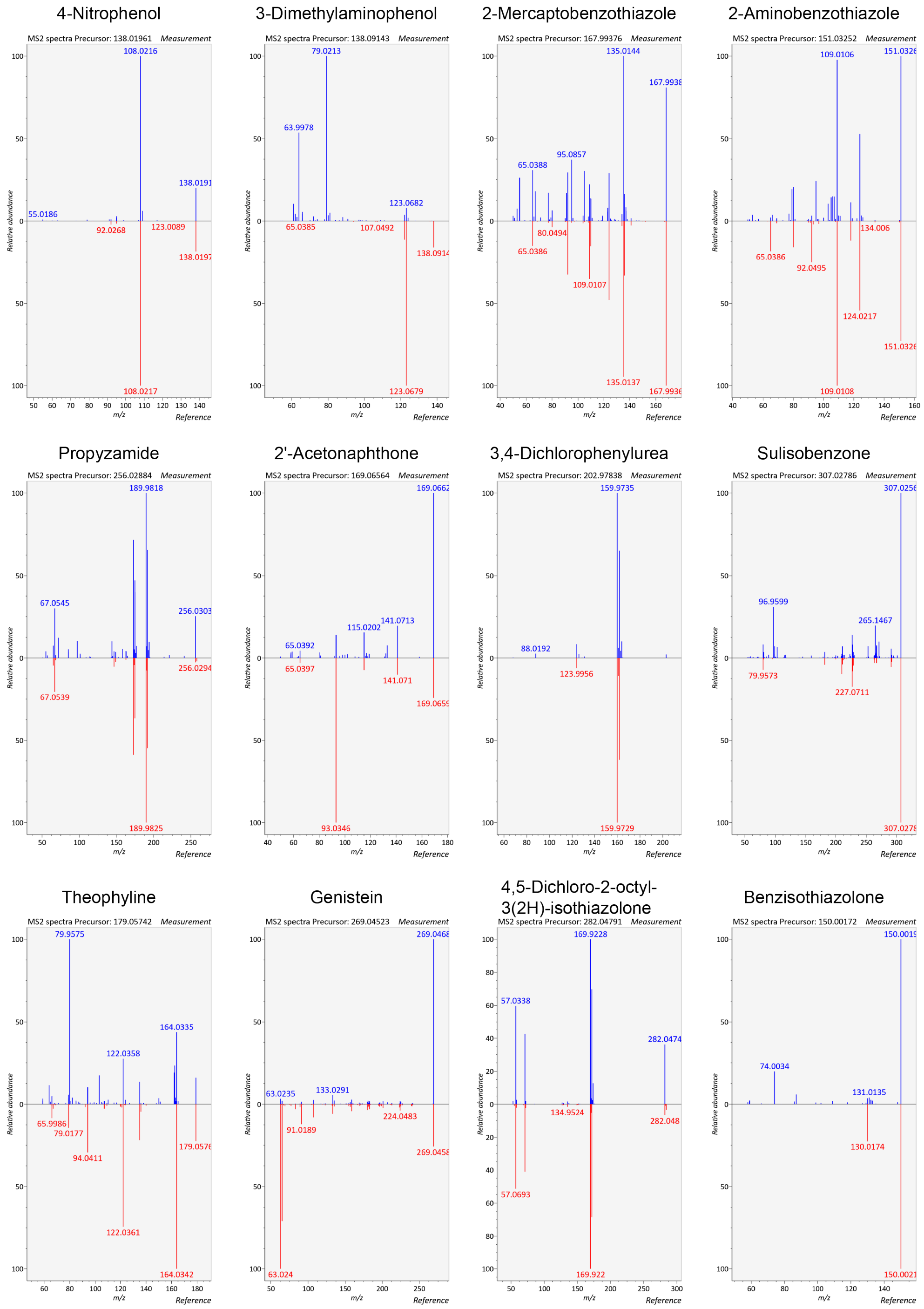


Figure SF17: MS2 spectra (blue, top) for the NTS features associated with toxicity matched to MassBank records (red, bottom).

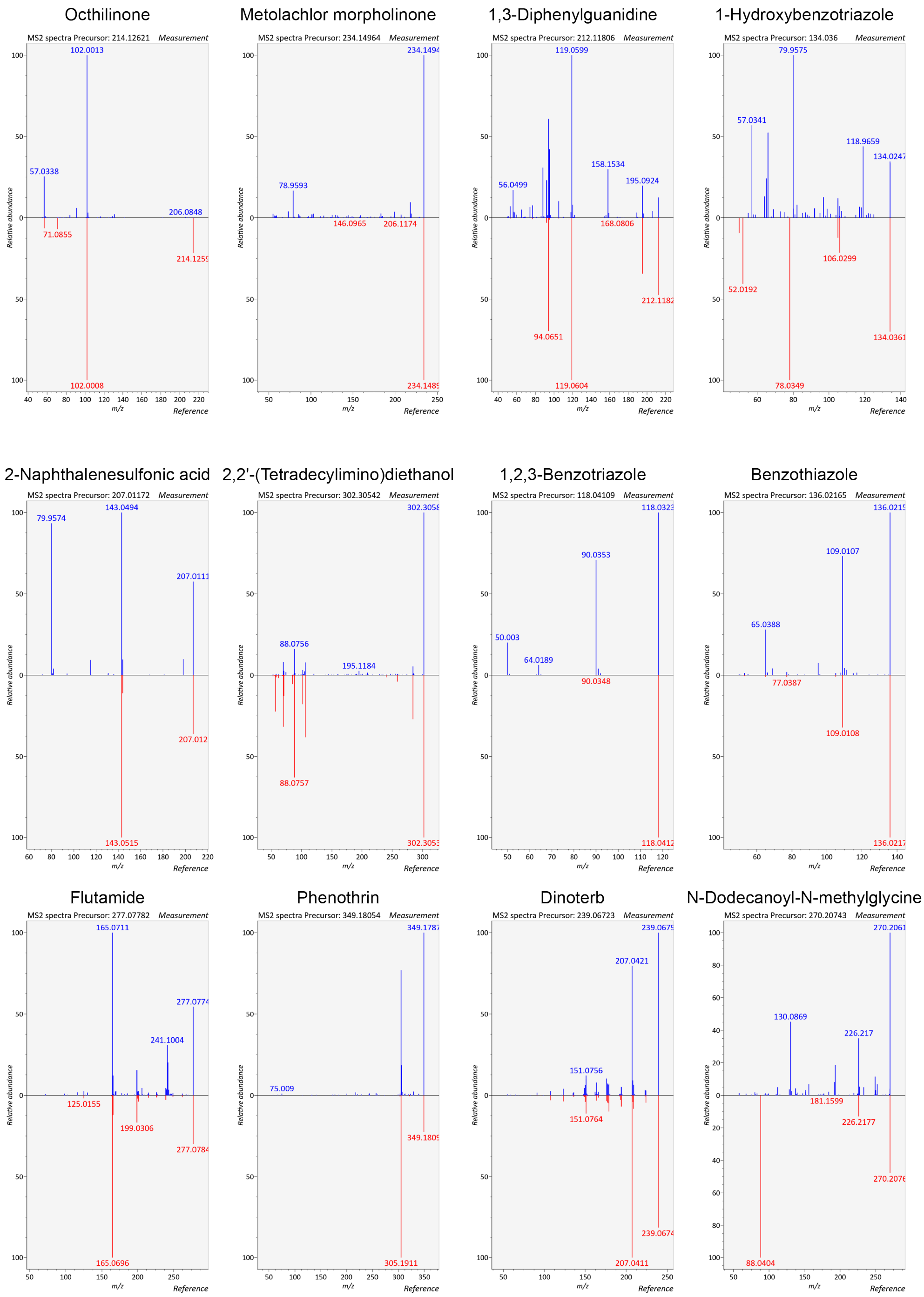


Figure SF18: MS2 spectra (blue, top) for the NTS features associated with toxicity matched to MassBank records (red, bottom).

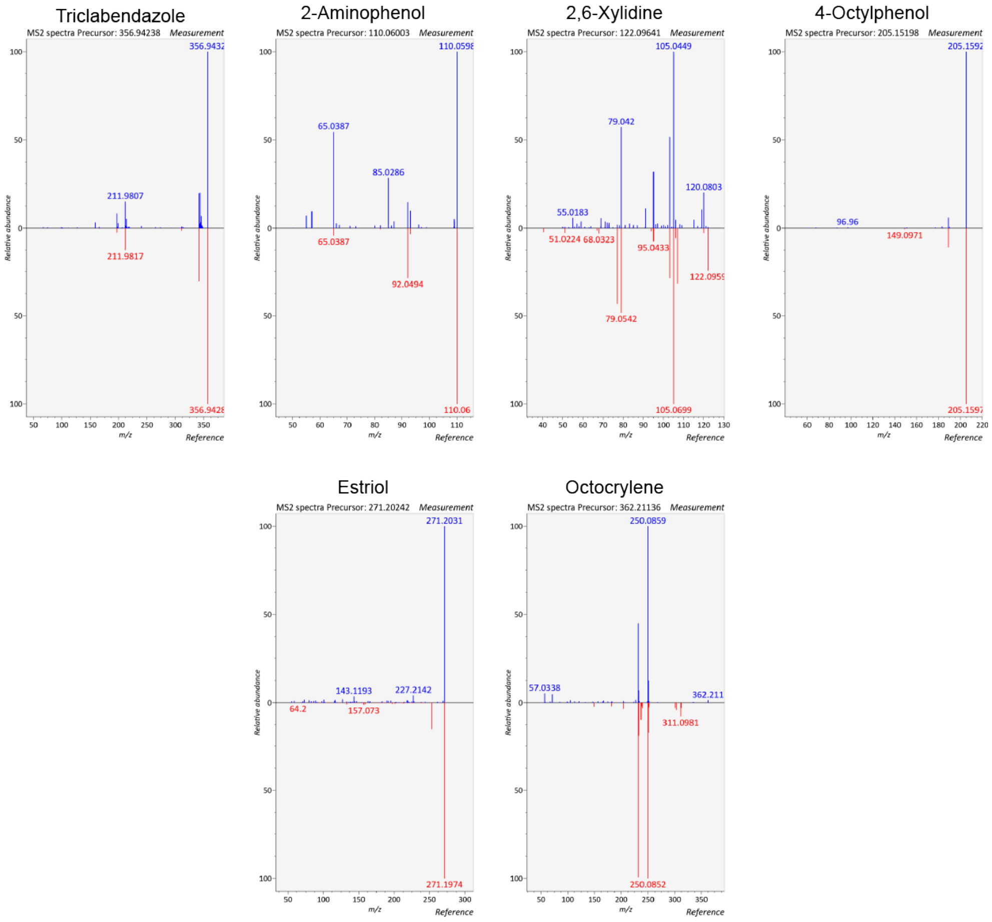


Figure SF19: MS2 spectra (blue, top) for the NTS features associated with toxicity matched to MassBank records (red, bottom).

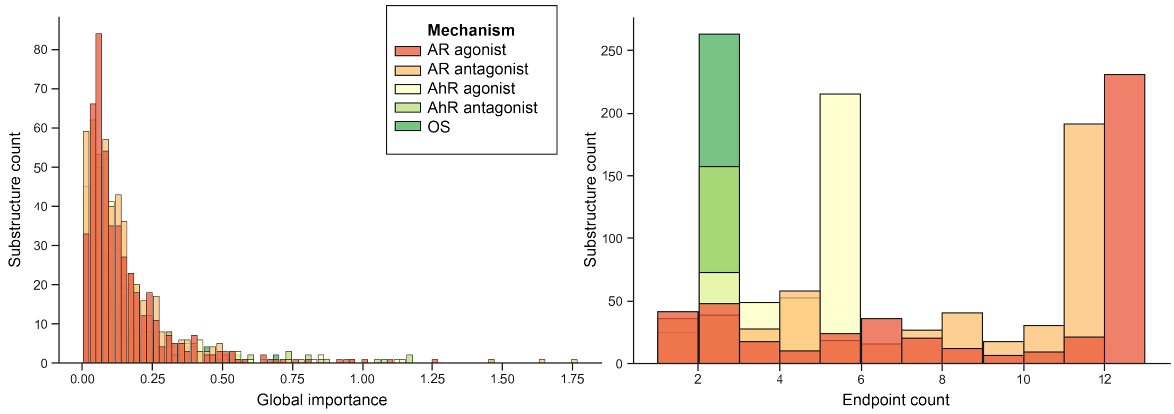


Figure SF20: Distribution of Global Feature Importance (left) and substructure recurrence (right) per mechanistic target.

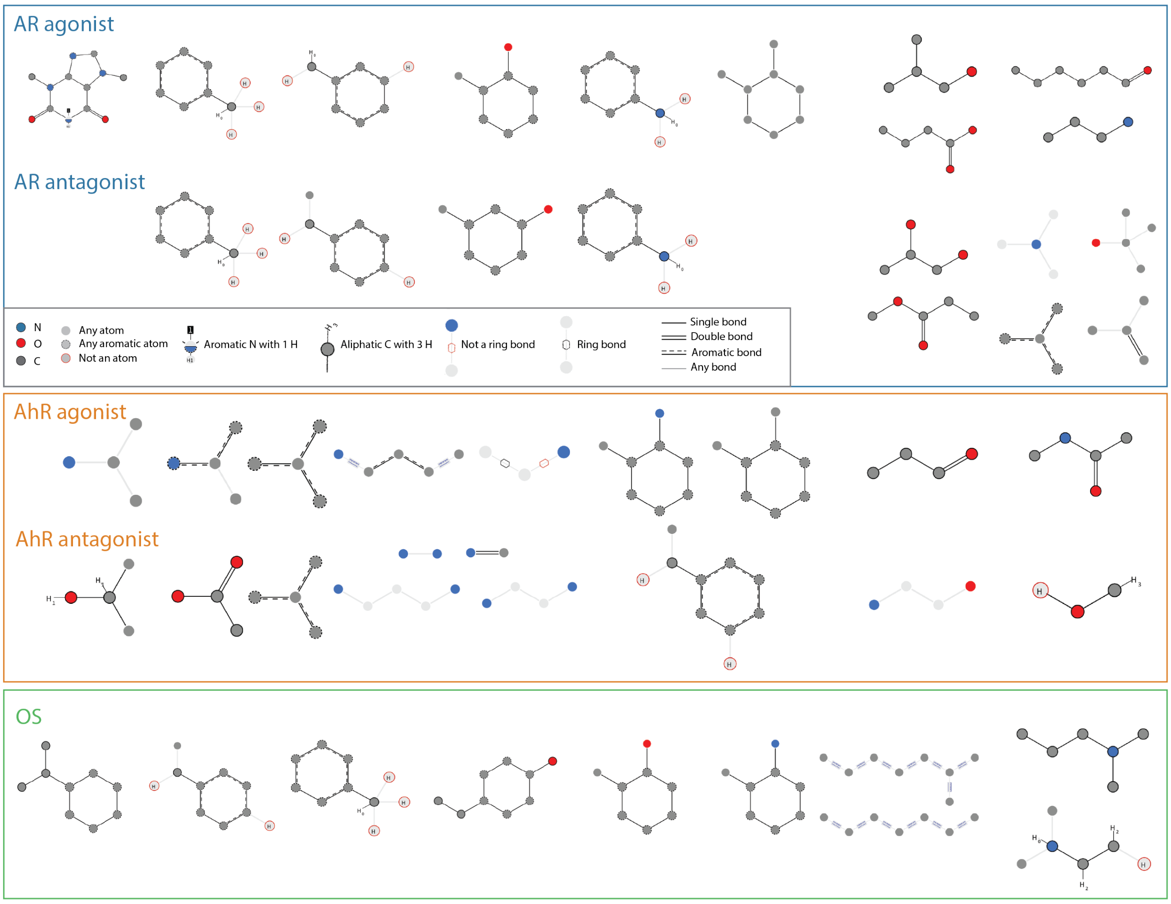


Figure SF21: Top 10 most important structural moieties for the prediction of AR (agonist and antagonist), AhR (agonist and antagonist), and OSR. The SMARTS patterns identified as important were visualized with SMARTS.plus package.[36] The legend applies to all three effects. A substructure had to be present in more than 75% of the endpoints for one mechanistic target and have a global importance value above 0.75 to be included. Thus, only structures tagged as important by independent models based on different endpoints were considered relevant, providing more generalisability.

# References

(1) Rotroff, D. M.; Dix, D. J.; Houck, K. A.; Kavlock, R. J.; Knudsen, T. B.; Martin, M. T.; Reif, D. M.; Richard, A. M.; Sipes, N. S.; Abassi, Y. A.; Jin, C.; Stampfl, M.; Judson, R. S. Real-time growth kinetics measuring hormone mimicry for ToxCast chemicals in T-47D human ductal carcinoma cells. *Chemical Research in Toxicology* **2013**, *26*, 1097–1107.

(2) Shah, I.; Setzer, R. W.; Jack, J.; Houck, K. A.; Judson, R. S.; Knudsen, T. B.; Liu, J.; Martin, M. T.; Reif, D. M.; Richard, A. M.; Thomas, R. S.; Crofton, K. M.; Dix, D. J.; Kavlock, R. J. Using ToxCast data to reconstruct dynamic cell state trajectories and estimate toxicological points of departure. *Environmental Health Perspectives* **2016**, *124*, 910–919.

(3) Medvedev, A.; Moeser, M.; Medvedeva, L.; Martsen, E.; Granick, A.; Raines, L.; Zeng, M.; Makarov Jr, S.; Houck, K. A.; Makarov, S. S. Evaluating biological activity of compounds by transcription factor activity profiling. *Science Advances* **2018**, *4*, eaar4666.

(4) Berg, E. L.; Kunkel, E. J.; Hytopoulos, E.; Plavec, I. Characterization of compound mechanisms and secondary activities by BioMAP analysis. *Journal of Pharmacological and Toxicological methods* **2006**, *53*, 67–74.

(5) Kleinstreuer, N. C.; Yang, J.; Berg, E. L.; Knudsen, T. B.; Richard, A. M.; Martin, M. T.; Reif, D. M.; Judson, R. S.; Polokoff, M.; Dix, D. J.; Kavlock, R. J.; Houck, K. A. Phenotypic screening of the ToxCast chemical library to classify toxic and therapeutic mechanisms. *Nature Biotechnology* **2014**, *32*, 583–591.

(6) Sipes, N. S.; Martin, M. T.; Kothiya, P.; Reif, D. M.; Judson, R. S.; Richard, A. M.; Houck, K. A.; Dix, D. J.; Kavlock, R. J.; Knudsen, T. B. Profiling 976 ToxCast chemicals across 331 enzymatic and receptor signaling assays. *Chemical Research in Toxicology* **2013**, *26*, 878–895.

(7) Zurlinden, T. J.; Saili, K. S.; Rush, N.; Kothiya, P.; Judson, R. S.; Houck, K. A.; Hunter, E. S.; Baker, N. C.; Palmer, J. A.; Thomas, R. S.; Knudsen, T. B. Profiling the ToxCast library with a pluripotent human (H9) stem cell line-based biomarker assay for developmental toxicity. *Toxicological Sciences* **2020**, *174*, 189–209.

(8) Paul Friedman, K.; Watt, E. D.; Hornung, M. W.; Hedge, J. M.; Judson, R. S.; Crofton, K. M.; Houck, K. A.; Simmons, S. O. Tiered high-throughput screening approach to identify thyroperoxidase inhibitors within the ToxCast phase I and II chemical libraries. *Toxicological Sciences* **2016**, *151*, 160–180.

(9) Wages, P. A.; Joshi, P.; Tallman, K. A.; Kim, H.-Y. H.; Bowman, A. B.; Porter, N. A. Screening ToxCast for Chemicals That Affect Cholesterol Biosynthesis: Studies in Cell Culture and Human Induced Pluripotent Stem Cell–Derived Neuroprogenitors. *Environmental Health Perspectives* **2020**, *128*, 017014.

(10) Judson, R. S.; Houck, K. A.; Kavlock, R. J.; Knudsen, T. B.; Martin, M. T.; Mortensen, H. M.; Reif, D. M.; Rotroff, D. M.; Shah, I.; Richard, A. M.; Dix, D. J. In vitro screening of environmental chemicals for targeted testing prioritization: the ToxCast project. *Environmental Health Perspectives* **2010**, *118*, 485–492.

(11) Judson, R. et al. Editor's highlight: Analysis of the effects of cell stress and cytotoxicity on in vitro assay activity across a diverse chemical and assay space. *Toxicological Sciences* **2016**, *152*, 323–339.

(12) Paul Friedman, K.; Gagne, M.; Loo, L.-H.; Karamertzanis, P.; Netzeva, T.; Sobanski, T.; Franzosa, J. A.; Richard, A. M.; Lougee, R. R.; Gissi, A. Utility of in vitro bioactivity as a lower bound estimate of in vivo adverse effect levels and in risk-based prioritization. *Toxicological Sciences* **2020**, *173*, 202–225.

(13) Berthold, M. R.; Cebron, N.; Dill, F.; Gabriel, T. R.; Kötter, T.; Meinl, T.; Ohl, P.; Thiel, K.; Wiswedel, B. KNIME - the Konstanz Information Miner: Version 2.0 and Beyond. *SIGKDD Explor. Newsl.* **2009**, *11*, 26–31.

(14) Watt, E. D.; Judson, R. S. Uncertainty quantification in ToxCast high throughput screening. *PLoS One* **2018**, *13*, e0196963.

(15) Cereto-Massagué, A.; Ojeda, M. J.; Valls, C.; Mulero, M.; Garcia-Vallvé, S.; Pujadas, G. Molecular fingerprint similarity search in virtual screening. *Methods* **2015**, *71*, 58–63.

(16) Durant, J. L.; Leland, B. A.; Henry, D. R.; Nourse, J. G. Reoptimization of MDL keys for use in drug discovery. *Journal of Chemical Information and Computer Sciences* **2002**, *42*, 1273–1280.

(17) PubChem, PubChem Substructure Fingerprint. Accessed Oct. 2019.

(18) Kier, L. B.; Hall, L. H. An electrotopological-state index for atoms in molecules. *Pharmaceutical Research* **1990**, *7*, 801–807.

(19) Klekota, J.; Roth, F. P. Chemical substructures that enrich for biological activity. *Bioinformatics* **2008**, *24*, 2518–2525.

(20) O'Boyle, N. M.; Banck, M.; James, C. A.; Morley, C.; Vandermeersch, T.; Hutchison, G. R. Open Babel: An open chemical toolbox. *Journal of Cheminformatics* **2011**, *3*, 1–14.

(21) Muegge, I.; Mukherjee, P. An overview of molecular fingerprint similarity search in virtual screening. *Expert opinion on drug discovery* **2016**, *11*, 137–148.

(22) Capecchi, A.; Probst, D.; Reymond, J.-L. One molecular fingerprint to rule them all: drugs, biomolecules, and the metabolome. *Journal of cheminformatics* **2020**, *12*, 1–15.

(23) Steinbeck, C.; Han, Y.; Kuhn, S.; Horlacher, O.; Luttmann, E.; Willighagen, E. The Chemistry Development Kit (CDK): An open-source Java library for chemo-and bioinformatics. *Journal of Chemical Information and Computer Sciences* **2003**, *43*, 493–500.

(24) Landrum, G. Rdkit documentation. *Release* **2013**, *1*, 4.

(25) Cao, D.-S.; Xu, Q.-S.; Hu, Q.-N.; Liang, Y.-Z. ChemoPy: freely available python package for computational biology and chemoinformatics. *Bioinformatics* **2013**, *29*, 1092–1094.

(26) Yap, C. W. PaDEL-descriptor: An open source software to calculate molecular descriptors and fingerprints. *Journal of Computational Chemistry* **2011**, *32*, 1466–1474.

(27) O'Boyle, N. M.; Morley, C.; Hutchison, G. R. Pybel: a Python wrapper for the OpenBabel cheminformatics toolkit. *Chemistry Central Journal* **2008**, *2*, 1–7.

(28) Hoyt, C. T.; Konotopez, A.; Ebeling, C. PyBEL: a computational framework for Biological Expression Language. *Bioinformatics* **2018**, *34*, 703–704.

(29) Mahmoud, R. S.; Yousef, A. H. Using molecular fingerprints as descriptors in toxicity prediction: a survey. 2019 IEEE International Conference on Bioinformatics and Biomedicine (BIBM). 2019; pp 2649–2654.

(30) Yang, C.; Tarkhov, A.; Marusczyk, J.; Bienfait, B.; Gasteiger, J.; Kleinoeder, T.; Magdziarz, T.; Sacher, O.; Schwab, C. H.; Schwoebel, J.; Terfloth, L.; Arvidson, K.; Richard, A.; Worth, A.; Rathman, J. New publicly available chemical query language, CSRML, to support chemotype representations for application to data mining and modeling. *Journal of Chemical Information and Modeling* **2015**, *55*, 510–528.

(31) Wang, J.; Hallinger, D. R.; Murr, A. S.; Buckalew, A. R.; Lougee, R. R.; Richard, A. M.; Laws, S. C.; Stoker, T. E. High-throughput screening and chemotype-enrichment analysis of ToxCast phase II chemicals evaluated for human sodium-iodide symporter (NIS) inhibition. *Environment International* **2019**, *126*, 377–386.

(32) Kosnik, M. B.; Strickland, J. D.; Marvel, S. W.; Wallis, D. J.; Wallace, K.; Richard, A. M.; Reif, D. M.; Shafer, T. J. Concentration–response evaluation of ToxCast compounds for multivariate activity patterns of neural network function. *Archives of Toxicology* **2020**, *94*, 469–484.

(33) Nelms, M. D.; Lougee, R.; Roberts, D. W.; Richard, A.; Patlewicz, G. Comparing and contrasting the coverage of publicly available structural alerts for protein binding. *Computational Toxicology* **2019**, *12*, 100100.

(34) Nyffeler, J.; Willis, C.; Lougee, R.; Richard, A.; Paul-Friedman, K.; Harrill, J. A. Bioactivity screening of environmental chemicals using imaging-based high-throughput phenotypic profiling. *Toxicology and Applied Pharmacology* **2020**, *389*, 114876.

(35) Neale, P. A.; Munz, N. A.; Ait-Aissa, S.; Altenburger, R.; Brion, F.; Busch, W.; Escher, B. I.; Hilscherová, K.; Kienle, C.; Novák, J. Integrating chemical analysis and bioanalysis to evaluate the contribution of wastewater effluent on the micropollutant burden in small streams. *Science of the Total Environment* **2017**, *576*, 785–795.

(36) Ehrt, C.; Krause, B.; Schmidt, R.; Ehmki, E. S.; Rarey, M. SMARTS.plus–A Toolbox for Chemical Pattern Design. *Molecular Informatics* **2020**, *39*, 2000216.
